# Rapid blood acid–base regulation by European sea bass (*Dicentrarchus labrax*) in response to sudden exposure to high environmental CO_2_

**DOI:** 10.1242/jeb.242735

**Published:** 2022-01-26

**Authors:** Daniel W. Montgomery, Garfield T. Kwan, William G. Davison, Jennifer Finlay, Alex Berry, Stephen D. Simpson, Georg H. Engelhard, Silvana N. R. Birchenough, Martin Tresguerres, Rod W. Wilson

**Affiliations:** 1 Biosciences, Geoffrey Pope Building, University of Exeter, Exeter, EX4 4QD, UK; 2 Marine Biology Research Division, Scripps Institution of Oceanography, University of California San Diego, 9500 Gilman Drive, La Jolla, CA 92093, USA; 3 National Oceanic and Atmospheric Administration Fisheries Service, Southwest Fisheries Science Center, 8901 La Jolla Shores Drive, La Jolla, CA 92037, USA; 4 Centre for Environment, Fisheries & Aquaculture Science (Cefas), Pakefield Road, Lowestoft, NR33 0HT, UK; 5 School of Environmental Sciences, University of East Anglia, Norwich, NR4 7TJ, UK

**Keywords:** Hypercapnia, Ionocytes, Respiratory acidosis, O_2_ transport, Gill plasticity

## Abstract

Fish in coastal ecosystems can be exposed to acute variations in CO_2_ of between 0.2 and 1 kPa CO_2_ (2000–10,000 µatm). Coping with this environmental challenge will depend on the ability to rapidly compensate for the internal acid–base disturbance caused by sudden exposure to high environmental CO_2_ (blood and tissue acidosis); however, studies about the speed of acid–base regulatory responses in marine fish are scarce. We observed that upon sudden exposure to ∼1 kPa CO_2_, European sea bass (*Dicentrarchus labrax*) completely regulate erythrocyte intracellular pH within ∼40 min, thus restoring haemoglobin–O_2_ affinity to pre-exposure levels. Moreover, blood pH returned to normal levels within ∼2 h, which is one of the fastest acid–base recoveries documented in any fish. This was achieved via a large upregulation of net acid excretion and accumulation of HCO_3_^−^ in blood, which increased from ∼4 to ∼22 mmol l^−1^. While the abundance and intracellular localisation of gill Na^+^/K^+^-ATPase (NKA) and Na^+^/H^+^ exchanger 3 (NHE3) remained unchanged, the apical surface area of acid-excreting gill ionocytes doubled. This constitutes a novel mechanism for rapidly increasing acid excretion during sudden blood acidosis. Rapid acid–base regulation was completely prevented when the same high CO_2_ exposure occurred in seawater with experimentally reduced HCO_3_^−^ and pH, probably because reduced environmental pH inhibited gill H^+^ excretion via NHE3. The rapid and robust acid–base regulatory responses identified will enable European sea bass to maintain physiological performance during large and sudden CO_2_ fluctuations that naturally occur in coastal environments.

## Abstract (precis)

**Summary:** European sea bass exposed to 1 kPa (10,000 µatm) CO_2_ regulate blood and red cell pH within 2 h and 40 min, respectively, protecting O_2_ transport capacity, via enhanced gill acid excretion.

## INTRODUCTION

Increased CO_2_ in aquatic environments, or environmental hypercapnia, causes significant physiological challenges for water-breathing animals including fish. As environmental CO_2_ increases, there is a corresponding rise in CO_2_ within the fish's blood, which in turn induces a decrease in blood pH (Brauner et al., 2019; Cameron and Randall, 1972). This condition is referred to as respiratory acidosis and, depending on its magnitude, can disrupt multiple homeostatic processes including gas exchange (Crocker and Cech, 1998; Eddy et al., 1977; Perry and Kinkead, 1989) and cardiovascular function (Lee et al., 2003; Perry and McKendry, 2001; Perry et al., 1999). Globally, CO_2_ levels in the ocean are increasing as a result of anthropogenic climate change, and are predicted to reach ∼0.1 kPa (0.1% CO_2_, 1000 µatm) by 2100 under a ‘business as usual’ scenario (Orr et al., 2005; Doney et al., 2011; IPCC, 2014).

The increase in oceanic CO_2_ levels, known as ocean acidification (OA) (Doney et al., 2009), has renewed interest in acid–base regulatory mechanisms of aquatic organisms. However, coastal and estuarine environments already experience much larger variations in CO_2_ levels (Sunda and Cai, 2012; Wallace et al., 2014), which are likely to be exacerbated in the future (Cai et al., 2011; Melzner et al., 2013). High variation in *P*_CO_2__ within coastal environments primarily results from diel changes in metabolic activity (i.e. a switch between net autotrophy during the day and net heterotrophy at night) (Baumann et al., 2015; Saderne et al., 2013), riverine inputs (Salisbury et al., 2008), coastal upwelling events (Feely et al., 2008; Saderne et al., 2013) and increased impacts of anthropogenic activities (Cai et al., 2011; Duarte et al., 2013). Fluctuations in *P*_CO_2__* *can occur rapidly over minutes to hours, and reach levels greater than 10 times higher than atmospheric CO_2_ concentrations (>0.4 kPa CO_2_) (Baumann et al., 2015; Hofmann et al., 2011; Melzner et al., 2013). In addition, estuarine areas in European coastal waters have been documented to reach *P*_CO_2__ levels approaching 1 kPa (1% CO_2_, 10,000 µatm) (Borges et al., 2006), which may result in mobile species experiencing rapid changes in CO_2_ as they move through these habitats. This type of environmental hypercapnia implies a different physiological challenge to fish compared with OA. Firstly, as environmental CO_2_ levels increase above CO_2_ levels in venous blood of fish (typically ∼0.3 kPa), CO_2_ diffusion gradients are reversed, resulting in net uptake from the environment into the blood and inducing a much more pronounced respiratory acidosis (Tresguerres and Hamilton, 2017). Secondly, the sudden and extreme CO_2_ fluctuations must be met by equally fast, robust and reversible acid–base regulatory responses.

Fish have a great capacity to restore blood pH to compensate for CO_2_-induced respiratory acidosis, which is largely achieved by excreting excess H^+^ and absorbing HCO_3_^−^ using their gills (Brauner et al., 2019; Claiborne et al., 2002; Esbaugh, 2017; Evans et al., 2005; Perry and Gilmour, 2006). At the cellular level, these processes take place in specialised ion-transporting cells, or ionocytes. However, the underlying ion-transporting proteins and regulatory mechanisms are intrinsically different between freshwater and marine fishes and may also vary between species (Brauner and Baker, 2009; Claiborne et al., 2002; Evans et al., 2005; Perry and Gilmour, 2006). The few studies that have investigated acid–base regulation after acute exposure to ∼1 kPa CO_2_ in marine fish have reported a large variation of responses, with full blood pH compensation occurring between ∼2 and 24 h depending on the species (Hayashi et al., 2004; Larsen et al., 1997; Perry, 1982; Toews et al., 1983). Given the exquisite sensitivity of most proteins to changes in pH, variation in the time course of acid–base regulatory responses between species has important implications for whole-organism performance. Haemoglobins (Hb) of fish species show strong Bohr and Root effects (Wells, 2009) which reduce Hb–O_2_ binding affinity and the O_2_ carrying capacity when erythrocyte intracellular pH (pH_i_) decreases. While fish have adaptations to regulate pH_i_ of erythrocytes (Cossins and Richardson, 1985; Nikinmaa and Tufts, 1989; Thomas and Perry, 1992), erythrocyte pH_i_ in many fish species (particularly marine fish) is closely linked to whole-blood pH (Brauner and Baker, 2009; Shartau et al., 2020). Therefore, adaptations which enhance the speed of whole-blood acid–base regulation will also provide faster restoration of O_2_ transport capacity and minimise disruption to energetically expensive activities such as foraging and digestion. However, little is known about why some species are able to compensate for respiratory acidosis faster than others.

The gill ionocytes of marine fish excrete H^+^ using apical Na^+^/H^+^ exchangers (NHEs) driven by basolateral Na^+^/K^+^-ATPase (NKA) (Brauner et al., 2019; Claiborne et al., 2002; Evans et al., 2005). Theoretically, H^+^ excretion during sudden exposure to hypercapnia could be upregulated by increased biosynthesis of NKA and NHE; however, transcriptional and translational responses typically take at least a few hours (e.g. Tresguerres et al., 2005, 2006). Furthermore, protein turnover is energetically expensive (Pan et al., 2015), so short-term regulation of H^+^ excretion by synthesis and degradation of ion-transporting proteins would not be particularly efficient. Alternatively, the rapid upregulation of H^+^ excretion may be mediated by post-translational regulatory modifications such as insertion of pre-existing proteins into the ionocyte membrane, or morphological adjustments of its apical area, as reported for a variety of fishes in response to other acid–base disturbances (reviewed in Brauner et al., 2019; Tresguerres et al., 2019).

In the present study, we investigated acid–base regulation of European sea bass, *Dicentrarchus labrax*, an active predator which seasonally inhabits shallow coastal, estuarine and saltmarsh environments (Doyle et al., 2017) where rapid and large fluctuations in CO_2_ levels occur (Baumann et al., 2015). Specifically, we characterised blood acid–base regulation, erythrocyte pH_i_ and O_2_ affinity, effects of seawater chemistry on speed of acid–base regulation, and changes in ionocyte NKA and NHE3 abundance, intracellular localisation, and apical surface area during acute exposure to ∼1 kPa CO_2_.

## MATERIALS AND METHODS

### Capture and pre-experimental conditions

Juvenile European sea bass, *Dicentrarchus labrax* (Linnaeus 1758), were obtained by seine netting in estuaries and salt marshes from Dorset and the Isle of Wight on the south coast of the United Kingdom. Sea bass were transferred to the Aquatic Resources Centre of the University of Exeter where they were held in ∼500 l tanks in a recirculating aquaculture system (RAS, total volume ∼2500 l) at temperatures between 14 and 22°C. Sea bass were fed 3 times a week with commercial pellet (Horizon 80, Skretting) with a supplement of chopped frozen mussel (*Mytilus edulis*) once a week. For ∼6 months before experiments, sea bass were maintained at a temperature of 14°C and seawater CO_2_ levels of ∼0.05–0.06 kPa (pH ∼8.10). Prior to all experimental procedures, food was withheld from sea bass for a minimum of 72 h. Animal collections were conducted under appropriate permits (Marine Management Organisation permit no. 030/17 and Natural England permit no. OLD1002654) and all experimental procedures were carried out under UK Home Office licence P88687E07 and approved by the University of Exeter's Animal Welfare and Ethical Review Board.

### Hypercapnia exposure

Individual sea bass were moved to isolation tanks (∼20 l) and left to acclimate overnight for a minimum of 12 h before exposure to hypercapnia. During the acclimation period, isolation tanks were fed by the RAS at a rate of ∼4 l min^−1^; with overflowing water recirculated back to the RAS. After overnight acclimation, hypercapnia exposure began by switching the inflow of water from low CO_2_ control conditions to high CO_2_ seawater delivered from a header tank (∼150 l) in which *P*_CO_2__ levels had already been increased to ∼1 kPa using an Aqua Medic pH computer (AB Aqua Medic GmbH). The pH computer maintained header tank *P*_CO_2__ levels using an electronic solenoid valve which fed CO_2_ to a diffuser in the header tank if the pH rose above 6.92 and stopped CO_2_ flow if the pH dropped below 6.88. Additionally, to reduce CO_2_ fluctuation in isolation tanks during exposures, the gas aerating each tank was switched from ambient air to a gas mix of 1% CO_2_, 21% O_2_ and 78% N_2_ (G400 Gas mixing system, Qubit Biology Inc.). During exposures, overflowing water from each isolation tank recirculated to the header tank creating an isolated experimental system of ∼250 l. The experimental system was maintained at 14°C using a heater/chiller unit (Grant TX150 R2, Grant Instruments) attached to a temperature exchange coil in the header tank. To characterise the time course of acid–base regulation, sea bass were exposed to hypercapnia (∼1 kPa CO_2_) for ∼10, ∼40 or ∼135 min before measurements were taken. The pH of isolation tanks was monitored with a separate pH probe and matched the header tank ∼5 min after initial exposure. Measurements of an additional group of sea bass were obtained at normocapnic CO_2_ levels (∼0.05 kPa CO_2_) to act as a pre-exposure control (hereafter this group is referred to as time=0). At the time of sampling, measurements of seawater pH (NBS scale), temperature and salinity, as well as samples of seawater to measure total CO_2_ (TCO_2_)/dissolved inorganic carbon (DIC) were taken from each isolation tank. DIC analysis was conducted using a custom-built system described in detail by Lewis et al. (2013). Measurements of pH, salinity, temperature and DIC were then input into CO2SYS (Pierrot et al., 2006) to calculate *P*_CO_2__ and total alkalinity (TA) based on the equilibration constants refitted by Dickson and Millero (1987).

### Blood sampling and analysis

Following hypercapnia exposures (Table 1), sea bass were individually anaesthetised *in situ* with a dose of 100 mg l^−1^ benzocaine. Anaesthetic was introduced into the isolation box without any visual or physical disturbance to sea bass, thus eliminating capture or handling stress prior to sampling. When sea bass were moderately anaesthetised (no response to physical stimuli and cessation of gill ventilation), they were immediately transferred to a gill irrigation tank (containing 40 mg l^−1^ benzocaine to maintain anaesthesia) in which gill ventilation was artificially maintained by a micro-pump. Once gill water flow was stable (gill operculum just open and exhalant water flow just visualised), blood was sampled via caudal vessel puncture using a heparinised 1 ml syringe. The gill irrigation tank used was filled with water from the header tank and maintained at an appropriate *P*_CO_2__ level by aeration with the same gas mix feeding the isolation tanks. The water chemistry of the gill irrigation chamber was measured following the same procedures outlined for the isolation chambers, with one DIC sample taken at the end of blood sampling (Table S1).

**Table 1.**
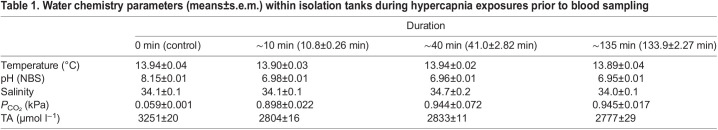
Water chemistry parameters (means±s.e.m.) within isolation tanks during hypercapnia exposures prior to blood sampling

Immediately after sampling, extracellular pH was measured on 30 µl of whole blood using an Accumet micro-pH electrode and Hanna HI8314 pH meter calibrated to 14°C using pH_NBS_ 7.04 and 9.21 appropriate buffers. Measurements of blood pH were made in a temperature-controlled water bath. Three 75 µl microcapillary tubes were then filled with whole blood and anaerobically sealed with Critoseal capillary tube sealant (Fisher) and paraffin oil, and centrifuged for 2 min at 10,000 rpm. Haematocrit (Hct) was measured using a Hawksley micro-haematocrit reader. Plasma was then extracted from capillary tubes for analysis of TCO_2_ using a Mettler Toledo 965D carbon dioxide analyser. Plasma *P*_CO_2__ and HCO_3_^−^ were then calculated from TCO_2_, temperature and blood pH using the Henderson–Hasselbalch equation with values for solubility and pK^1^_app_ based on Boutilier et al. (1984, 1985). The Hb content of 10 µl of whole blood was also assessed by the cyanmethaemoglobin method (after addition to 2.5 ml of Drabkin's reagent, Sigma). Half the remaining whole blood was centrifuged at 10,000 rpm for 2 min at 4°C. The resulting plasma was separated and 10 µl was diluted in ultrapure water, snap frozen in liquid N_2_, and stored at −80°C before later being used to measure plasma cation and anion concentrations using ion chromatography (Dionex ICS 1000 and 1100, Thermo Scientific, UK). The remaining plasma was snap frozen in liquid N_2_ and stored at −80°C before measurements of plasma lactate and glucose were made (YSI 2900D Biochemistry Analyzer, Xylem Analytics). After separating the plasma, the surface of the leftover erythrocyte pellet was blotted to remove the leukocyte layer. The erythrocyte pellet was then snap frozen in liquid nitrogen for 10 s and thawed in a 37°C water bath for 1 min prior to pH_i_ measurements as described by Zeidler and Kim (1977), and validated by Baker et al. (2009b). All measurements or storage of blood occurred within 10 min of blood sampling. Finally, the *P*_50_ of whole blood was measured as a proxy for Hb–O_2_ affinity following the methods outlined in Montgomery et al. (2019) using a Blood Oxygen Binding System (BOBS, Loligo Systems), as detailed by Oellermann et al. (2014). The *P*_50_ point is the *P*_O_2__ at which 50% of Hb is bound to O_2_ and a higher *P*_50_ indicates a right shift in the O_2_ dissociation curve of whole blood and a reduction in Hb–O_2_ affinity.

### Flux measurements

The flux of acid–base relevant ions between sea bass and seawater was measured over a ∼135 min time period in normocapnic conditions (*n*=7) and immediately following exposure to hypercapnia (*n*=8, Table 2). At the start of the measurements the flow to the isolation tanks was stopped and water chemistry maintained at the desired *P*_CO_2__ by gassing the tanks with either ambient air (control) or a 1% CO_2_ gas mix (hypercapnia). Seawater samples for measuring TA were taken at the beginning and end of the ∼135 min flux period, preserved by adding 40 µl of 4% (w/v) mercuric chloride per 10 ml of seawater, and stored at 4°C (Dickson et al., 2007) prior to analysis by double titration using an autotitrator (Metrohm 907 Titrando with 815 Robotic USB Sample Processor XL, Metrohm). TA measurements were made using a double titration method modified from Cooper et al. (2010) as detailed by Middlemiss et al. (2016). Briefly, 20 ml samples were titrated to pH 3.89 using 0.02 mol l^−1^ HCl whilst gassing with CO_2_-free N_2_; pH was then returned to starting values by titrating with 0.02 mol l^−1^ NaOH. Samples for measuring total ammonia were frozen at −20°C before ammonia concentration was measured using a modified version of the colorimetric method of Verdouw et al. (1978) at 660 nm using a microplate reader (NanoQuant infinite M200 pro, Tecan Life Sciences). A calibration curve was constructed using NH_4_Cl standards in seawater.

**Table 2.**
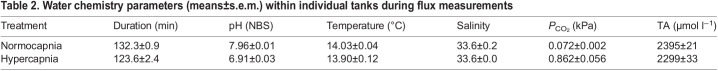
Water chemistry parameters (means±s.e.m.) within individual tanks during flux measurements

Acid–base relevant flux (µmol kg^−1^ h^−1^) was then calculated using the following equation:
(1)

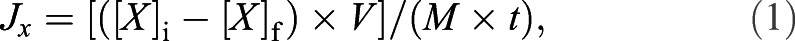
as described by Wilson and Grosell (2003), where *V* is the volume of water (l) in the isolation tank (after the initial sample is taken), *M* is the mass of the sea bass (kg), *t* is the duration of the flux period (h), and [*X*]_i_ and [*X*]_f_ are the ion concentrations in the chamber water (µmol l^−1^) at the beginning and end of the flux period, respectively. By reversing the initial and final values, titratable acid, instead of base, flux can be calculated so that a positive value equals acid uptake (i.e. HCO_3_^−^ excretion) and a negative value equals acid excretion (i.e. HCO_3_^−^ uptake). We then calculated net acid–base flux (µeq kg^−1^ h^−1^) as the sum of titratable acid and total ammonia (*T*_amm_) flux (McDonald and Wood, 1981).

### Exposure to low total alkalinity and pH

A group of sea bass was exposed to ∼1 kPa CO_2_ for ∼135 min in low total alkalinity and pH seawater in order to examine whether the mechanism for acid–base regulation was affected by these water chemistry parameters. Sufficient 1 mol l^−1^ HCl was added to ∼250 l seawater to reduce TA by over 90% from ∼2800 µmol l^−1^ to ∼200 µmol l^−1^, followed by overnight aeration to equilibrate CO_2_ with atmospheric levels. We then adjusted the *P*_CO_2__ of the low TA seawater to the desired level of ∼1 kPa as described above, and a pH set point of 5.75. Sea bass were placed in the individual isolation boxes (fed by the RAS as detailed for normal TA hypercapnia exposures) and left to acclimate overnight before being exposed to the combined low TA and hypercapnia treatment. Flow to individual isolation boxes was stopped, and ∼75% of the water from the isolation box was drained and refilled with low TA, hypercapnic water. This process was repeated 3 times over a period of ∼5 min. The gas mix aerating each isolation box was switched from ambient air to a 1% CO_2_ gas mix to maintain the desired *P*_CO_2__ levels. After ∼135 min exposure, each sea bass was anaesthetised and sampled for blood acid–base measurements as detailed previously. The water chemistry of isolation boxes and gill irrigation chambers was measured at the time of blood sampling (Table S2).

### Gill sampling

Gill tissue was sampled from sea bass exposed to ambient CO_2_ conditions (*n*=5) and to hypercapnia for ∼135 min (*n*=5, taken immediately after the flux measurements) in normal TA seawater (Table S3). Mean water chemistry conditions during flux measurements (Table 2) and those experienced by sea bass prior to gill sampling (Table S3) differ because gill samples were only collected from 5 of the 8 sea bass used for flux measurements. Sea bass were euthanised in an anaesthetic bath (benzocaine, 1 g l^−1^), and gills were dissected and rinsed in phosphate-buffered saline (PBS). The first gill arch on the left side was flash frozen in liquid N_2_ and stored at −80°C for Western blotting, and the first gill arch on the right side was fixed in 4% paraformaldehyde in 0.1 mol l^−1^ phosphate buffer saline (PBS) (diluted from 16% electron microscopy grade paraformaldehyde; Electron Microscope Science catalogue no. 15710), overnight at 4°C for immunohistochemistry. Following ∼10 h fixation, gill samples were transferred to 50% ethanol for ∼10 h at 4°C, and then stored in 70% ethanol at 4°C.

### Antibodies

NKA was immunodetected using α5, a mouse monoclonal antibody against the α-subunit of chicken NKA (a5, Developmental Studies Hybridoma Bank, Iowa City, IA, USA; Lebovitz et al., 1989). This antibody universally recognises NKA in teleost fishes including yellowfin tuna (*Thunnus albacares*; Kwan et al., 2019), Pacific chub mackerel (*Scomber japonicus*; Kwan et al., 2020) and California killifish (*Fundulus parvipinnis*; Nadler et al., 2021). Rabbit anti-NHE3 polyclonal antibodies were generously donated by Dr Junya Hiroi (St Marianna University School of Medicine, Kawaski, Japan); they target two epitope regions within rainbow trout (*Oncorhynchus mykiss*) NHE3b (GDEDFEFSEGDSASG and PSQRAQLRLPWTPSNLRRLAPL), and recognise NHE3 of multiple teleost species including European sea bass (*D. labrax*; Blondeau-Bidet et al., 2019). The secondary antibodies were goat anti-mouse HRP-linked and goat anti-rabbit HRP-linked (Bio-Rad, Hercules, CA, USA) for immunoblotting, and goat anti-mouse Alexa Fluor 546 and goat anti-rabbit Alexa Fluor 488 (Invitrogen, Grand Island, USA) for immunohistochemistry.

### Western Blotting

Western blotting followed the procedures outlined in Kwan et al. (2019, 2020). While frozen on dry ice, the gill filament and lamellae were separated from the gill arch using a razor blade. The excised tissue was then immersed in liquid N_2_ and pulverised in a porcelain grinder, then submerged within an ice-cold, protease-inhibiting buffer (250 mmol l^−1^ sucrose, 1 mmol l^−1^ EDTA, 30 mmol l^−1^ Tris, 10 mmol l^−1^ benzamidine hydrochloride hydrate, 1 mmol l^−1^ phenylmethanesulfonyl fluoride, 1 mmol l^−1^ dithiothreitol, pH 7.5). Samples were further homogenised using a handheld VWR Pellet Mixer (VWR, Radnor, PA, USA) for 15 s intervals (3 times) while on ice. Next, samples were centrifuged (3000 ***g***, 4°C; 10 min), and the resulting supernatant was considered the crude homogenate. An aliquot of the crude homogenate was further subjected to a higher speed centrifugation (21,130 ***g***, 4°C; 30 min), and the pellet was saved as the membrane-enriched fraction. The Bradford assay was used to determine protein concentration (Bradford, 1976), which was used to normalise protein loading.

On the day of Western blotting, samples were mixed with an equal volume of 90% 2× Laemmli buffer and 10% β-mercaptoethanol. Samples were then heated at 70°C for 5 min, and the proteins (20 µg per lane) were loaded onto a polyacrylamide mini gel (4% stacking, 10% separating) – alternating between control and high CO_2_ treatments to avoid possible gel lane effects. The gel ran at 60 V for 15 min, then 100 V for 1.5 h, and proteins were transferred onto a polyvinylidene difluoride (PVDF) membrane using a wet transfer cell (Bio-Rad) at 70 V for 2 h at 4°C. PVDF membranes were then incubated in Tris-buffered saline with 1% Tween (TBS-T) with milk powder (0.1 g ml^−1^) at room temperature for 1 h, then incubated with primary antibody (NKA: 10.5 ng ml^−1^; NHE3: 1:1000) in blocking buffer at 4°C overnight. The following day, PVDF membranes were washed in TBS-T (3 times; 10 min each), incubated in blocking buffer with secondary antibodies (1:10,000) at room temperature for 1 h, and washed again in TBS-T (3 times; 10 min each). Bands were made visible through addition of ECL Prime Western Blotting Detection Reagent (GE Healthcare, Waukesha, WI, USA) and imaged with the Universal III Hood (Bio-Rad). Following imaging, the PVDF membrane was incubated in Ponceau stain (10 min, room temperature) to estimate protein loading. Relative NKA and NHE protein abundance (*n*=5 per treatment) was quantified using the Image Lab software (version 6.0.1; Bio-Rad) and normalised by the protein content in each lane. The anti-NKA antibodies detected a single band of ∼100 kDa (Fig. S2, left), which matches previous reports from multiple fish species ranging from ∼90 to ∼120 kDa (Chandrasekar et al., 2014; Dutta et al., 2019; Frommel et al., 2021; Kwan et al., 2020, 2021; McCormick et al., 2009). The anti-NHE3 antibodies detected a single band of ∼70 kDa (Fig. S2, right), which is smaller than what may be expected based on bioinformatic predictions for NHE3s from other animals. The discrepancy between predicted and apparent NKA and NHE3 sizes may be explained by intrinsic limitations in Western blotting for precisely determining protein size (Bass et al., 2017; Gwozdz and Dorey, 2017), together with the presence of post-translational modifications and isoforms (see Harter et al., 2021, for a case of a fish NHE with an alternative start codon that results in a smaller-than-predicted protein). For example, the predicted size of NHE3 is 97 kDa in climbing perch *Anabas testudineus*, 94 kDa in killifish *Fundulus heteroclitus* and 93 kDa in rabbit *Oryctolagus cuniculus*, yet their apparent Western blot sizes are ∼85 kDa (Chen et al., 2017), ∼77 kDa (Edwards et al., 2005) and ∼80 kDa (Biemesderfer et al., 1997), respectively. Although many studies have previously used the same anti-NHE3 antibodies as us for immunohistochemistry in other fish species (Blondeau-Bidet et al., 2019; Christensen et al., 2012; Hiroi and McCormick, 2012; Seo et al., 2013; Wright et al., 2016), to our knowledge only Wright et al. (2016) performed Western blots and did not report the predicted size of the immunoreactive band. Regardless, the presence of single Western blot bands close to previously reported protein sizes combined with the labelling of expected subcellular localisations of specific gill cells (see below) indicates that the anti-NKA and anti-NHE3 antibodies used in the current study specifically recognise their target proteins.

### Immunohistochemistry

Immunohistochemistry was performed as described in Kwan et al., (2020). Fixed gill tissue stored in 70% ethanol was rehydrated in PBS+0.1% Tween (PBS-T) for 10 min, and gill filaments were dissected out to ease subsequent imaging. Autofluorescence was quenched by rinsing in ice-cold PBS with sodium borohydride (1.0–1.5 mg ml^−1^; 6 times; 10 min each), followed by incubation in blocking buffer (PBS-T, 0.02% normal goat serum, 0.0002% keyhole limpet haemocyanin) at room temperature for 1 h. Samples were incubated with blocking buffer containing primary antibodies (NKA: 40 ng ml^−1^; NHE3: 1:500, cf. Seo et al., 2013) at 4°C overnight. The following day, samples were washed in PBS-T (3 times at room temperature; 10 min each), and incubated with the fluorescent secondary antibodies (1:500) counterstained with DAPI (1 µg ml^−1^) at room temperature for 1 h. Samples were washed again in PBS-T as before, then placed on a concave slide for imaging using an inverted confocal microscope (Zeiss LSM 800 with Zeiss ZEN 2.6 blue edition software; Cambridge, UK). Samples incubated without primary antibodies had no signal (Fig. S1A).

### Quantification of ionocyte apical surface area

The apical surface area of gill ionocytes were quantified through a combination of whole-mount imaging (40× objective lens with deionised water immersion), optical sectioning, and *XZ*- and *YZ*-plane analysis. Gill filaments immunostained for NKA and NHE3 were coded, then blind analysis was employed to eliminate experimental bias. A relatively flat surface on the gill filament was selected under 0.5× scanning confocal magnification to ensure imaging would be performed on ionocytes in an upright position, thus minimising errors in apical surface area quantification due to angle distortion. After locating an ionocyte by its distinctive NKA signal, the scanning confocal magnification was increased to 5.0× and the entire cell was *Z*-stack imaged (optimal interval automatically selected: 0.07–0.12 µm per slice). Subsequent viewing of the *Z*-stack from the *X–Z* and *Y–Z* planes allowed us to assess intracellular localisation, and to identify the image slice that captured the entire apical surface (typically, the second slice from the top of the cell). Next, the ionocyte apical surface area (identified by NHE3 immunofluorescence signal) was quantified using FIJI (Schindelin et al., 2012). The apical surface area for control and hypercapnia-exposed fish (*n*=5) was determined as the mean apical surface area of five ionocytes taken from different gill filaments.

### Statistical analysis

All statistical analysis was performed using R v3.6.3 (http://www.R-project.org/). Changes in blood chemistry parameters over time in response to hypercapnia exposure were analysed using one-way ANOVA before assumptions of equal variance of data and normality of model residuals were checked. *Post hoc* tests were conducted on least-square means generated by package ‘emmeans’ (https://CRAN.R-project.org/package=emmeans), with Tukey adjusted *P*-values for multiple comparisons. Some data did not meet required assumptions for one-way ANOVA. Unequal variances were observed in measurements of plasma *P*_CO_2__ between treatments; as such, we used Welch's ANOVA with Tukey's pairwise comparisons using Benjamini–Hochberg corrections for *post hoc* testing. Measurements of blood pH and *P*_50_ did not meet assumptions of normality and were analysed using the Kruskal–Wallis test with *post hoc* comparisons made with Dunn's test from package ‘FSA’ (https://github.com/droglenc/FSA), using Benjamini–Hochberg corrections for multiple comparisons. As a result of unusually high measurements of plasma [Cl^−^] and [Na^+^] in some samples, a ROUT test was conducted (*Q*=0.5%) in Graphpad Prism 9 to identify potential outliers (Motulsky and Brown, 2006). Samples in which plasma [Cl^−^] and [Na^+^] were both identified as outliers (*n*=6) by the ROUT test were excluded from the dataset prior to subsequent statistical analysis. The relationship between plasma [Cl^−^] and [HCO_3_^−^] as well as plasma [Na^+^] and [H^+^] across treatments was examined using Kendall's tau correlation. Comparisons of blood chemistry between fish exposed to hypercapnia for ∼135 min in normal alkalinity and low total alkalinity (and pH) seawater were made using two-sample *t*-tests. Measurements of plasma HCO_3_^−^ were analysed using Welch's *t*-test as unequal variances were observed between fish exposed to hypercapnia in normal and low total alkalinity seawater. Flux measurements were analysed using Student's *t*-test after checking data met assumptions of normality and equal variance. Relative protein abundance and ionocyte apical area met both assumptions of normality and equal variance and were analysed using one-tailed *t*-test (control response<CO_2_-exposed response).

## RESULTS

### Blood chemistry

**Fig. 1.**
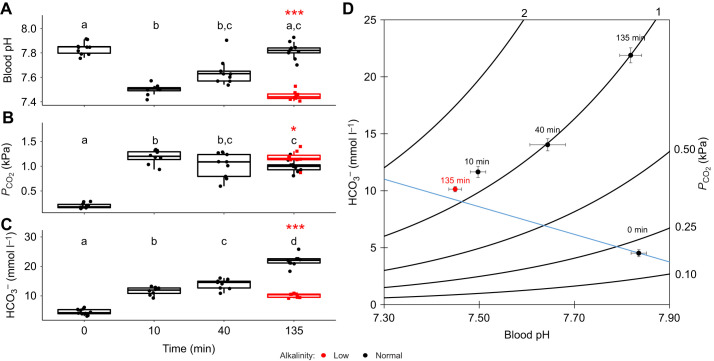
**Changes in blood chemistry between European sea bass in control conditions and after exposure to ∼0.9 kPa CO_2_ in normal alkalinity and low alkalinity seawater.** Fish were exposed to control conditions [∼0.05 kPa CO_2_, total alkalinity (TA) ∼2800 µmol l^−1^, time=0, *n*=10], or ∼0.9 kPa CO_2_ for ∼10 min (*n*=8), ∼40 min (*n*=9) and ∼135 min (*n*=9) in normal alkalinity (TA ∼2800 µmol l^−1^) seawater and ∼135 min in low alkalinity (TA ∼200 µmol l^−1^) seawater. (A) Blood pH, (B) plasma *P*_CO_2__ and (C) plasma HCO_3_^−^. Box plots show median (horizontal line), upper and lower quartiles (box) and 1.5× interquartile range (whiskers); circles are individual data points. Significant differences between control fish and fish exposed to ∼0.9 kPa CO_2_ in normal TA conditions are indicated by different lowercase letters at each time point (A: Dunn's test, *P*<0.05; B: pairwise comparison using Benjamini–Hochberg correction, *P*<0.05; C: pairwise comparison of least square means, *P*<0.05). Significant differences between sea bass exposed to ∼0.9 kPa CO_2_ for ∼135 min in normal TA seawater (black) and low TA seawater (red) are indicated by asterisks (*P<0.05 or ***P<0.001) (A: two-sample *t*-test, *P*<0.001; B: two-sample *t*-test, *P*<0.05; C: Welch's *t*-test, *P*<0.001). (D) Combined changes of all three acid–base parameters are expressed as a pH/HCO_3_^−^/*P*_CO_2__ diagram for sea bass in normal TA and low TA at different time points (blue dashed line indicates estimated non-bicarbonate blood buffer line based on equations from Wood et al., 1982). Values represent means±s.e.m.

Exposure to environmental hypercapnia caused significant changes in blood pH of sea bass over time (Kruskal–Wallis test, χ^2^=25.0, d.f.=3, *P*<0.001). There was a pronounced acidosis of the blood from pH 7.84±0.02 (mean±s.e.m.) in control conditions (normocapnia, time=0) to 7.50±0.03 after exposure to hypercapnia for ∼10 min (Fig. 1A,D). Following this initial acidosis, sea bass completely restored blood pH to control levels after ∼135 min (Fig. 1A,D). Blood pH regulation was accompanied by a ∼5-fold increase in plasma HCO_3_^−^, from 4.5±0.3 to 21.9±0.7 mmol l^−1^, over the ∼135 min exposure (Fig. 1C,D; one-way ANOVA, *F*=203.3, d.f.=3, *P*<0.001).

Plasma *P*_CO_2__ showed significant changes during exposure to hypercapnia (Fig. 1B,D; Welch's ANOVA, *F*=202.5, d.f.=3, *P*<0.001). The initial decrease in blood pH of sea bass was driven by a rapid and large (∼6-fold) increase in plasma *P*_CO_2__, from 0.200±0.016 to 1.185±0.049 kPa CO_2_, within the first 10 min of exposure. There was a small but significant decline in plasma *P*_CO_2__ between sea bass sampled ∼10 min after exposure and sea bass sampled ∼135 min after exposure (Fig. 1B). There were no significant differences in plasma glucose or lactate levels between any treatment groups, with values for all sea bass of 5.90±0.43 mmol l^−1^ and 0.45±0.05 mmol l^−1^, respectively.

To test the influence of total alkalinity and environmental pH on acid–base regulation, a group of sea bass were exposed to hypercapnia for ∼135 min in low alkalinity and pH seawater. These sea bass were unable to compensate for a respiratory acidosis when exposed to acute hypercapnia for ∼135 min. Blood pH was 0.37 units (95% confidence interval, CI 0.33–0.41) lower than that of sea bass exposed to hypercapnia in normal alkalinity seawater for the same length of time (Fig. 1A; two-sample *t*-test, d.f.=15, *t*=−13.551, *P*<0.001). Additionally, sea bass in low alkalinity seawater did not actively accumulate HCO_3_^−^ when exposed to environmental hypercapnia for ∼135 min (Fig. 1C; Welch's *t*-test, d.f.=9.8996, t=−16.817, *P*<0.001). Indeed, the increase in mean plasma [HCO_3_^−^] followed the predicted non-bicarbonate buffering line (Fig. 1D).

### Flux measurements

**Fig. 2.**
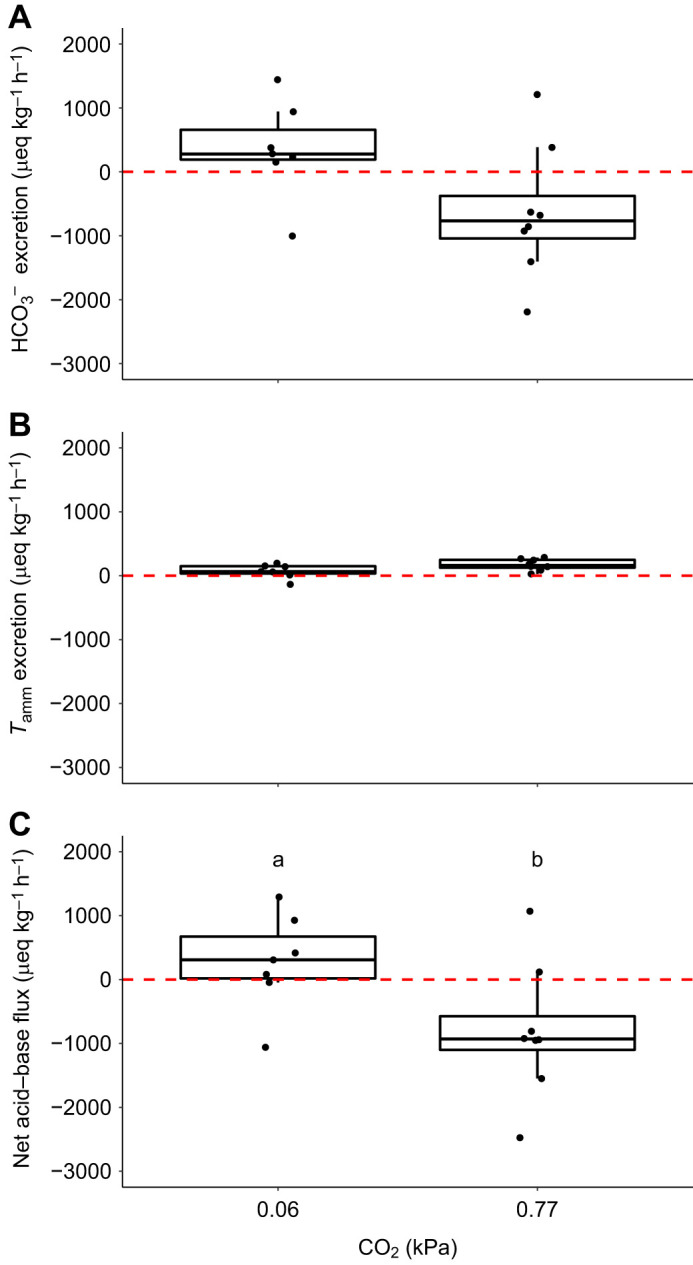
**Changes in flux measurements between European sea bass in control conditions and after ∼135 min exposure to hypercapnia.** Fish were exposed to control conditions (0.06 kPa CO_2_, *n*=7) or to ∼135 min hypercapnia (∼0.77 kPa CO_2_, *n*=8). (A) Excretion of HCO_3_^−^, (B) excretion of total ammonia (*T*_amm_) and (C) net acid–base flux. Significant differences in parameters are indicated by different lowercase letters (Student's *t*-test, *P*<0.05).

Sea bass switched from slight net base excretion under control normocapnic conditions to net acid excretion that was ∼2.5-fold larger in magnitude during 135 min of hypercapnia (Fig. 2C, Student's *t*-test, *t*=−2.25, d.f.=13, *P*=0.042). This was driven by a switch from a small apparent HCO_3_^−^ excretion to a large apparent HCO_3_^−^ uptake (Fig. 2A). There were no significant differences in *T*_amm_ excretion (Fig. 2B).

### Oxygen transport capacity

**Fig. 3.**
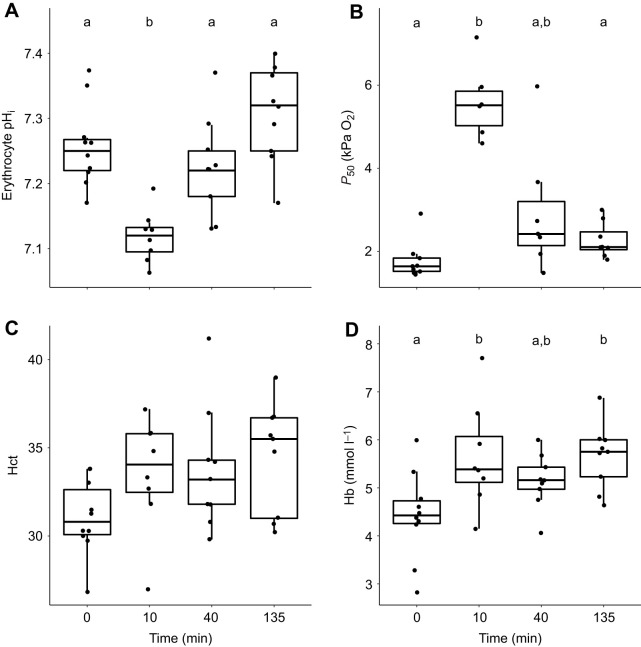
**Changes in oxygen transport capacity between European sea bass in control conditions (∼0.05 kPa CO_2_, time=0) and after exposure to ∼0.9 kPa CO_2_ for ∼10, ∼40 and ∼135 min.** (A) Erythrocyte intracellular pH (pH_i_), (B) haemoglobin (Hb)–O_2_ binding affinity (*P*_50_), (C) haematocrit (Hct) and (D) Hb level. Significant differences between parameters at each time point are indicated by different lowercase letters (A,C,D: pairwise comparisons of least square means, *P*<0.05; B: Dunn's test, *P*<0.05).

The initial drop in blood pH during hypercapnia exposure was reflected in changes in erythrocyte pH_i_ (Fig. 3A; one-way ANOVA, *F*=12.34, d.f.=3, *P*<0.001). However, erythrocyte pH_i_ returned to control levels after ∼40 min of exposure to hypercapnia (Fig. 3A). As expected, the significant changes in erythrocyte pH_i_ affected Hb–O_2_ binding affinity, leading to a ∼3-fold increase in *P*_50_ after 10 min, from 1.78±0.30 kPa O_2_ in control sea bass to 5.60±0.36 kPa O_2_ (Kruskall–Wallis test, χ^2^=17.4, d.f.=3, *P*<0.001). The rapid recovery of erythrocyte pH_i_ after ∼40 min led to *P*_50_ returning to pre-exposure levels (Fig. 3B).

Sea bass exposed to hypercapnia also experienced a ∼25% increase in Hb levels (Fig. 3D), at ∼10 min and∼135 min compared with control sea bass (one-way ANOVA, *F*=4.60, d.f.=3, *P*=0.009). In addition, sea bass exposed to hypercapnia exhibited an ∼8–10% increase in Hct (Fig. 3C), although this increase was marginally non-significant (one-way ANOVA, *F*=2.40, d.f.=3, 0.086).

### Plasma ion concentrations

**Fig. 4.**
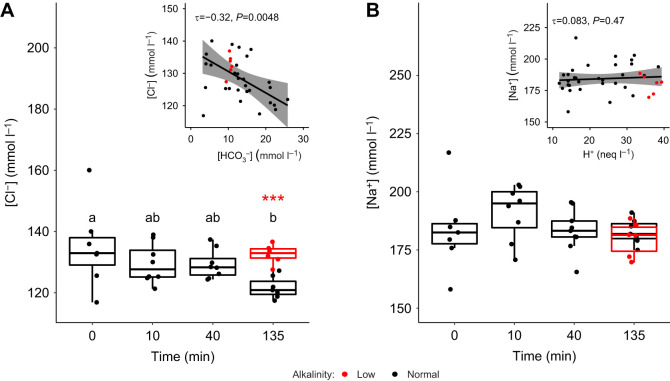
**Comparison of plasma ion concentrations between European sea bass in control conditions and exposed to hypercapnia in normal or low TA seawater.** Fish were exposed to control conditions (*n*=7, time=0), or hypercapnia for ∼10 min (*n*=8), ∼40 min (*n*=9) or ∼135 min (*n*=7) in normal (∼2800 µmol l^−1^) TA seawater or for ∼135 min in low (∼200 µmol l^−1^) TA seawater (*n*=6). (A) Plasma [Cl^−^] and (B) plasma [Na^+^]. Significant differences between [Cl^−^] in sea bass in normal TA seawater at each time point are indicated by different lowercase letters (pairwise comparison of least squares means, *P*<0.05). The significant difference between [Cl^−^] in sea bass exposed to hypercapnia for ∼135 min in normal TA and low TA seawater is indicated by asterisks (two sample *t*-test, ***P<0.001). No significant differences were observed in [Na^+^]. Insets in A and B show correlation between plasma [Cl^−^] and [HCO_3_^−^] and between plasma [Na^+^] and [H^+^], respectively. τ and *P*-values represent results of Kendall's tau correlation. Shaded area represents 95% confidence interval (CI) of linear regression between measures. For insets and measurements taken after ∼135 min of exposure to hypercapnia, the colour indicates the TA treatment (i.e. black, normal TA; red, low TA).

Plasma [Cl^−^] significantly decreased by 13.1 mmol l^−1^ (95% CI 10.1–16.2 mmol l^−1^) from 134.9±5.1 mmol l^−1^ (mean±s.e.m.) in sea bass in normocapnia to 121.7±1.3 mmol l^−1^ in sea bass exposed to hypercapnia for ∼135 min (Kruskall–Wallis test, χ^2^=7.87, d.f.=3, *P*=0.049). Sea bass exposed to hypercapnia in low alkalinity seawater had significantly higher plasma [Cl^−^] when compared with fish in normal alkalinity seawater (Fig. 4A; two sample *t*-test, d.f.=11, *t*=5.7306, *P*<0.001). Decreases in plasma [Cl^−^] showed a correlation with increasing bicarbonate (Fig. 4A inset; Kendall's tau correlation, τ=−0.32, *P*=0.005). Plasma [Na^+^] showed no significant changes over the time course of hypercapnia exposure (one-way ANOVA, *F*=1.063, *P*=0.391), and there were no differences in [Na^+^] after ∼135 min of exposure to hypercapnia between sea bass in normal and low TA seawater (Fig. 4B; two-sample *t*-test, d.f.=11, *t*=−0.84857, *P*=0.414). As such, there was no correlation between plasma [Na^+^] and [H^+^] (Fig. 4B inset; Kendall's tau correlation, τ=0.083, *P*=0.47).

### NKA and NHE3 protein abundance

Exposure to hypercapnia did not induce significant changes in the abundance of NKA or NHE3 in crude homogenates (indicative of total protein abundance) or the abundance of NKA and NHE3 in membrane-enriched fractions (indicative of protein that was present in the apical or basolateral plasma membranes) (one-tailed *t*-test, *P*>0.05; Table 3).

**Table 3.**
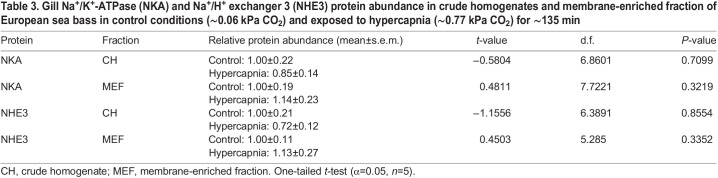
Gill Na^+^/K^+^-ATPase (NKA) and Na^+^/H^+^ exchanger 3 (NHE3) protein abundance in crude homogenates and membrane-enriched fraction of European sea bass in control conditions (∼0.06 kPa CO_2_) and exposed to hypercapnia (∼0.77 kPa CO_2_) for ∼135 min

### Ionocyte intracellular localisation and apical surface area

**Fig. 5.**
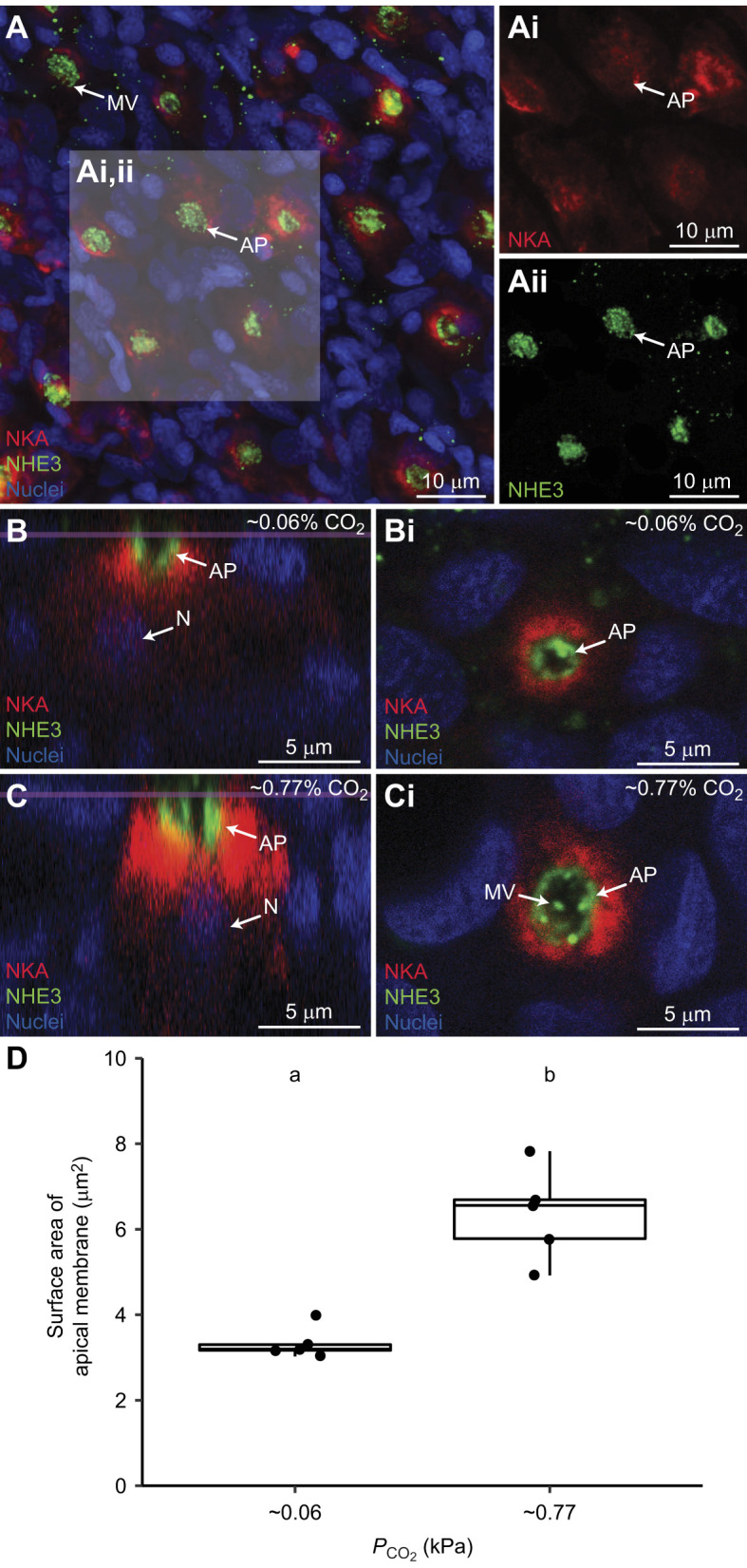
**Comparison of Na^+^/K^+^-ATPase (NKA) and Na^+^/H^+^ exchanger 3 (NHE3A) protein levels in European sea bass in control conditions and exposed to hypercapnia.** (A) European sea bass gill ionocytes express abundant basolateral NKA (Ai, red) and apical NHE3 (Aii, green). (B–D) Comparison of gill ionocytes between fish exposed to control conditions (∼0.06 kPa CO_2_; B) and ∼0.77 kPa CO_2_ for ∼135 min (C) revealed no changes in intracellular localisation, but hypercapnia-exposed fish had significantly wider apical surface area (D) (*n*=5 per treatment, one-tailed *t*-test, *P*=0.001). The purple line in B and C denotes the slice at which Bi and Ci were imaged. Nuclei (blue) are stained with DAPI. MV, microvilli; AP, apical pit; N, nuclei.

NKA-rich ionocytes were primarily localised on the gill filament trailing edges and the basal portion of the gill lamellae (Fig. S1B); all NKA-rich ionocytes also expressed NHE3 in their apical region (Fig. 5A). Despite analysis using high magnification imaging, optical sectioning and *X–Z* and *Y–Z* plane visualisation, we found no evidence of NHE3 intracellular localisation (Fig. 5B,Bi,C,Ci). Blind analysis revealed the ionocyte's apical surface area (based on the NHE3 signal) significantly increased, almost doubling from 3.34±0.17 to 6.36±0.49 µm^2^, after exposure to ∼135 min of hypercapnia (one-tailed *t*-test, *t*=5.873, d.f.=4.963, *P*=0.001; Fig. 5D).

## DISCUSSION

Our results indicate that European sea bass are able to rapidly compensate for hypercapnia-induced blood acidosis when the environmental CO_2_ is at the extreme high end of the spectrum encountered in their natural habitat. Complete restoration of blood pH after exposure to ∼1 kPa CO_2_ was achieved within ∼2 h via a switch from net base excretion to net acid excretion and a subsequent accumulation of HCO_3_^−^ in plasma. In addition, erythrocyte pH_i_ and Hb–O_2_ affinity were restored to pre-exposure levels after just ∼40 min, and there was a 20% increase in the blood Hb concentration together with a trend for ∼10% Hct increase. These results suggest an adrenergic response that stimulates a β-NHE in the erythrocytes (Nikinmaa, 2012), and contracts the spleen, resulting in the release of erythrocytes into the circulation (Crocker and Cech, 1997; Lee et al., 2003; Perry and Kinkead, 1989; Vermette and Perry, 1988). The end result is a boost in blood O_2_ transport capacity that counteracts the reduced Hb–O_2_ affinity induced by the initial hypercapnia-induced acidosis.

Regulation of respiratory acidosis by sea bass exposed to hypercapnia in normal alkalinity sea water (TA ∼2800 µmol l^−1^) resulted in an elevation of plasma [HCO_3_^−^] by ∼18 mmol l^−1^, which was correlated with a decrease in plasma [Cl^−^] of ∼13 mmol l^−1^. In comparison, while we saw a slight rise in plasma [Na^+^] on initial exposure to hypercapnia, this was transient, and there was no overall correlation between plasma [Na^+^] and [H^+^] during the whole 135 min experiment. However, a lack of increase in plasma [Na^+^] during acid–base regulation does not preclude increased Na^+^ uptake (to facilitate H^+^ excretion) during acid–base regulation. Instead, a lack of increased plasma [Na^+^] may simply reflect upregulation of the hypo-ionoregulatory mechanism for NaCl excretion in marine teleosts, which depends on transcellular Cl^−^ movement via basolateral Na^+^/K^+^/2Cl^−^ cotransporters and apical Cl^−^ channels, and on paracellular Na^+^ movement (Zadunaisky, 1996). In principle, this could compensate for the enhanced uptake of Na^+^ into the blood that is associated with H^+^ excretion by NHE, and would also help explain the observed reduction in plasma [Cl^−^] in fish exposed to hypercapnia.

In comparison, sea bass exposed to hypercapnia in low alkalinity sea water (TA ∼200 µmol l^−1^) showed no ability to accumulate HCO_3_^−^, to compensate for respiratory acidosis, and did not experience a decrease in plasma [Cl^−^]. At face value, these results may support potential direct uptake of HCO_3_^−^ from seawater in exchange for blood Cl^−^ through HCO_3_^−^/Cl^−^ exchange across the gills (Esbaugh et al., 2012; Perry and Gilmour, 2006; Tovey and Brauner, 2018). However, the thermodynamics of this proposed mechanism are not clear, as [Cl^−^] is much higher in seawater than in internal fluids of fish, and the opposite is true for [HCO_3_^−^]. This implies that both counterions would have to be transported against their concentration gradients and, furthermore, these gradients would become increasingly unfavourable as blood acidosis is compensated for. Importantly, our hypercapnic low alkalinity seawater had a pH of ∼5.7, which was ∼1.2 pH units lower than hypercapnic normal alkalinity seawater (a 15-fold increase in [H^+^]). Based on nominal values of [Na^+^] and [H^+^] inside fish gill ionocytes and calculations in Parks et al. (2008), the low alkalinity seawater would not sustain H^+^ excretion via apical NHEs (Table S4). Interestingly, if the NHE3 is not activated, there would not be an associated increased Na^+^ load into the blood and, as Na^+^ excretion is coupled to Cl^−^ excretion as explained above, there would be no need to upregulate NaCl excretion, potentially explaining the observed lack of decrease of plasma [Cl^−^]. Overall, we believe this evidence supports enhanced NHE3-mediated H^+^ excretion and associated retention of metabolically produced HCO_3_^−^ in the blood as the primary mechanism underlying regulation of respiratory acidosis in sea bass. However, we cannot exclude a potential role for Cl^−^-dependent HCO_3_^−^ absorption from sea water as an additional mechanism for acid–base compensation by sea bass. Evidence from some other teleost species after exposure to equivalent levels of environmental CO_2_ show changes in gene expression of ionocyte transporters and carbonic anhydrase, which may lead to more favourable conditions for apical HCO_3_^−^ uptake (Esbaugh et al., 2012; Tseng et al., 2013). Unfortunately, experiments to produce unequivocal evidence to distinguish between apical H^+^ excretion and HCO_3_^−^ uptake during acid–base regulation are not trivial because of the interlink and reciprocal compensation between ion-transporting processes for acid–base and ionoregulation in marine fish gills and intestine.

Immunostaining confirmed the presence of basolateral NKA and apical NHE3 in European sea bass gill ionocytes, indicating these cells play a role in H^+^ excretion. Moreover, all NKA-rich cells co-expressed NHE3. Noteworthy, a recent study reported a lack of NH3 immunostaining in gills of seawater-acclimated *D. labrax* (Blondeau-Bidet et al., 2019). This is puzzling because they used the same species, similar environmental salinity and the same antibodies as in our study. Additionally, that study was able to detect NHE3 mRNA in gills from the same specimens. We do not know the reasons for this discrepancy between positive gene expression but a lack of immunostaining for the protein itself, but we speculate this might be due to differences between *D. labrax* populations, feeding and exercise regimes, immunostaining protocols, or reagents, potentially coupled with a disconnect between mRNA and protein expression.

To investigate the mechanisms used by sea bass to enhance acid excretion, we examined whether changes in gill NKA and NHE3 occur after acute (∼135 min) exposure to hypercapnia. Gill NKA and NHE3 protein abundance did not change, ruling out increased protein synthesis as the mechanism responsible for the observed upregulation in acid excretion; this is not surprising considering the short time frame of our experiments. We also examined the potential translocation of pre-existing NKA and NHE3 to the ionocyte basolateral and apical membranes, respectively. Such mechanisms upregulate acid–base regulatory ion transport in elasmobranchs (Roa et al., 2014; Tresguerres et al., 2005, 2006, 2007b) and hagfish (Parks et al., 2007; Tresguerres et al., 2007a); however, NKA and NHE3 protein abundance in the gill membrane fraction of European sea bass gills was also unchanged, ruling out NKA and NHE3 translocation in our experiments. Finally, we hypothesised that sea bass could have remodelled the apical membrane of ionocytes to increase the sites for H^+^ excretion in contact with seawater. Indeed, this was the case as the surface area of the NHE3-abundant apical membrane of NKA-rich ionocytes roughly doubled in response to hypercapnia. Based on the accepted model of acid excretion in marine fish gill ionocytes (Brauner et al., 2019; Claiborne et al., 2002; Evans et al., 2005), the mechanism probably also includes enhanced carbonic anhydrase activity as well as decreased HCO_3_^−^ excretion via apical anion exchangers and H^+^ absorption into the blood via basolateral NHE1. Given the velocity of the response (∼2 h), these changes in activity are likely to be mediated by changes in the availability of CO_2_ substrate and post-translational modifications rather than changes in protein abundance. Interestingly, marine Japanese flounder (*Paralichthys olivaceus*) exposed to 1 kPa CO_2_ for 24 h also showed evidence for an approximately doubling in gill ionocyte apical surface area (although the differences with control fish were not statistically significant, possibly as a result of the large and variable responses, as seen in fish exposed to 5 kPa CO_2_, which were part of the same analyses even though many of them died between 24 and 72 h after hypercapnic exposure) (Hayashi et al., 2013). Moreover, gill NKA activity remained unchanged upon 24 h exposure to 1 kPa CO_2_, suggesting that, like European sea bass, Japanese flounder are able to cope with this level of hypercapnia without having to upregulate the biosynthesis of ion-transporting proteins. Additional mechanisms for acid–base regulation, which were not measured in this study, may also contribute to compensation of acute respiratory acidosis in marine fish. For example, hyperventilation by red drum (*Sciaenops ocellatus*) in response to a 4 h exposure to 0.5 kPa CO_2_ has been estimated to reduce the need for metabolic compensation by ∼39% as a result of lower blood *P*_CO_2__ (Ern and Esbaugh, 2016). Although blood sampling in our study involves forced gill ventilation, potentially eliminating the ventilatory responses observed by Ern and Esbaugh (2016), we believe it is unlikely that the rapid acid–base regulation we observed in sea bass is an artefact of this sampling method. Indeed, it has previously been demonstrated that this method provides equivalent blood acid–base measurements to those for cannulated fish in which natural ventilatory responses would be present (see fig. 6 in Brauner et al., 2019).

Previous studies on freshwater fishes have also documented morphological adjustments in gill ionocytes upon comparable hypercapnic exposures. However, the responses were the opposite to our study, as those freshwater fishes experienced a significant reduction in ionocyte apical surface area (Baker et al., 2009a; Goss et al., 1992b; Leino and McCormick, 1984). In some cases, the apical membrane retracted into a more pronounced apical pit (Goss et al., 1992a, 1994), which was suggested to create a microenvironment with higher [Na^+^] compared with the bulk freshwater and to facilitate Na^+^/H^+^ exchange (Kumai and Perry, 2012). In contrast, exposure to more pronounced hypercapnia (8 kPa CO_2_ over 4 days) induced an increase in gill ionocyte apical surface area in freshwater catfish (*Ictalurus punctatus*) (Cameron and Iwama, 1987). This response was similar to that in the sea bass in our study; however, the considerably longer exposures and higher *P*_CO_2__ levels probably resulted in additional acid–base responses that were not investigated, such as increased synthesis of ion-transporting proteins, or a change in the H^+^ excreting mechanism (e.g. NHE versus V-type H^+^-ATPase). Regardless, the ability of sea bass to rapidly compensate for a blood respiratory acidosis by simply increasing gill ionocyte apical surface area is in large part possible as a result of the overabundance of Na^+^ in sea water, which establishes favourable conditions for NHE-mediated H^+^ excretion.

**Fig. 6.**
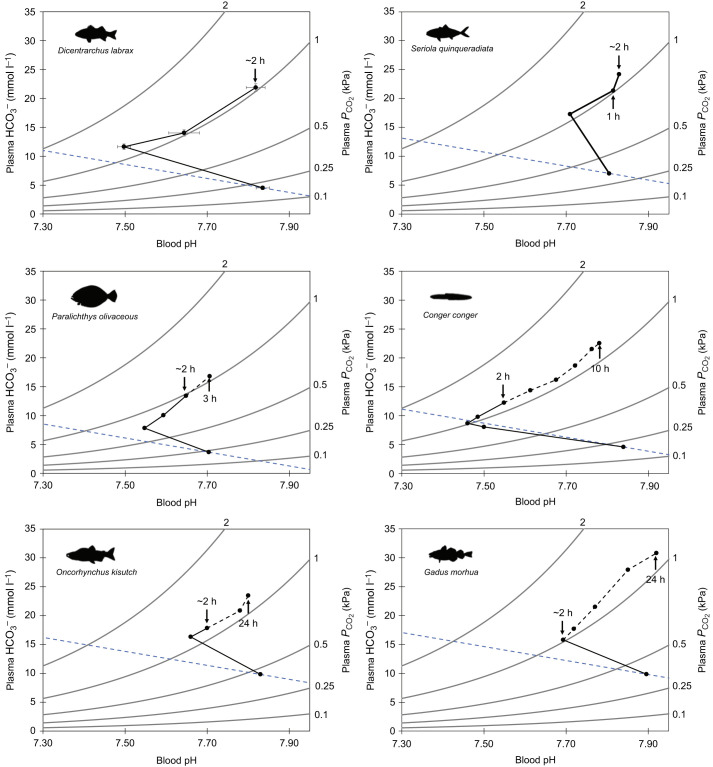
**Comparison of blood pH/HCO_3_^−^/*P*_CO_2__ across marine teleost species.** (A) European sea bass, *Dicentrarchus labrax* (present study), (B) Japanese amberjack, *Seriola quinqeradiata* (Hayashi et al., 2004; re-plotted raw data provided by personal communication with Dr Atsushi Ishimatsu, Can Tho University), (C) Japanese flounder, *Paralichthys olivaceus* (Hayashi et al., 2004), (D) conger eel, *Conger* (Toews et al., 1983), (E) coho salmon, *Oncorhynchus kisutch* (Perry, 1982), and (F) Atlantic cod, *Gadus morhua* (Larsen et al., 1997). The corresponding blood pH and HCO_3_^−^ of each species ∼2 h after 1 kPa CO_2_ exposure is indicated to allow direct comparisons with European sea bass. Times below the relevant point indicate when blood pH was not statistically different from pre-exposure levels for each species. The time course of the acid–base response after 2 h is indicated by a dashed black line. The dashed blue line is an approximated non-HCO_3_^−^ buffer line based on the mean Hct of sea bass from the present study and calculated using the equation for rainbow trout from Wood et al. (1982).

Freshwater species typically take from 24 h to >72 h to regulate blood pH after exposure to 1 kPa CO_2_ (Baker et al., 2009a; Claiborne and Heisler, 1984, 1986; Damsgaard et al., 2015; Larsen and Jensen, 1997; Perry, 1982; Perry et al., 1981; Smatresk and Cameron, 1982). While it is generally believed that marine teleosts can regulate their blood acid–base status at a faster rate than freshwater species (Brauner et al., 2019), relatively little research has been conducted to characterise the speed of acid–base regulation in marine fish. A bibliography search revealed four previous studies on five marine teleost species that characterised the time course of the acid–base regulatory response after exposure to 1 kPa CO_2_ (Fig. 6). Of these species, only the Japanese amberjack (*Seriola quinqueradiata*) was able to restore blood extracellular pH (pH_e_) faster than sea bass (∼60 min versus ∼135 min; Fig. 6B). The remaining four species regulated blood pH_e_ between 3 and 24 h post-CO_2_ exposure (Fig. 6C–F).

The robust ability of sea bass to rapidly acid–base regulate in response hypercapnia probably plays a significant role in their natural environment. Specifically, sea bass feed in shallow coastal estuaries and salt marsh habitats during the summer (Doyle et al., 2017), and these habitats typically experience large fluctuations in CO_2_ levels over short time periods (Hofmann et al., 2011; Melzner et al., 2013; Wallace et al., 2014). For example, equivalent salt marshes on the US coast regularly experience CO_2_ fluctuations of ∼0.4 kPa across tide cycles during the summer (Baumann et al., 2015). We should note that natural CO_2_ levels equivalent to those used in our study (∼1 kPa) have previously only been reported in upper estuaries where salinity is reduced (Borges et al., 2006) and high CO_2_ levels in estuaries often negatively correlate with salinity (Frankignoulle and Borges, 2001). Future studies should examine the impacts of salinity on acid–base regulation in euryhaline marine fish which may encounter acute hypercapnia in combination with reduced salinity. Despite this, the fast acid–base regulatory response observed in our study indicates that sea bass would be able to rapidly correct the respiratory blood acidosis caused by environmentally relevant, acute CO_2_ variation in seawater in <1 h. Critically, regulation of erythrocyte pH_i_ by sea bass occurred quicker than regulation of blood pH_e_. By rapidly restoring erythrocyte pH_i_, O_2_ transport capacity is maintained, which is crucial for active predatory teleosts. However, environmental CO_2_ variation cannot be the sole driver for enhanced acid–base regulatory capacities in all species. For example, Japanese amberjack show a similarly fast blood acid–base regulatory response (Hayashi et al., 2004) but primarily inhabit pelagic, offshore ecosystems in which large variation in environmental CO_2_ would be less likely to occur. Alternatively, it may be that active, predatory species have developed higher capacities for acid–base regulation to deal with large metabolic acidosis (as a result of anaerobic respiration used during intense exercise involved in prey capture). Understanding the mechanisms that determine species-specific differences in acid–base regulatory capacity will help us to understand the differential impacts of acute exposure to elevated CO_2_, both by itself and in combination with other stressors such as hypoxia. For example, we have recently reported that sea bass showed enhanced hypoxia tolerance when exposed to progressive and environmentally relevant hypercapnia and hypoxia over a 6 h period (Montgomery et al., 2019). In contrast, European plaice (*Pleuronectes platessa*) and European flounder (*Platichthys flesus*) exposed to the same conditions showed reduced hypoxia tolerance (*P*_crit_), which was associated with reduced Hb–O_2_ affinity and O_2_ uptake resulting from an uncompensated respiratory acidosis (Rogers, 2015; D.W.M. and R.W.W., unpublished observations). Thus, species with more robust acid–base regulatory mechanisms may be more resilient to interactive effects between hypercapnia and hypoxia.

Overall, our study highlights the capacity of European sea bass to rapidly (2 h) regulate blood and erythrocyte acid–base status and O_2_ transport capacity upon exposure to a pronounced and sudden increase in environmental CO_2_ levels. The ability of sea bass to rapidly upregulate H^+^ excretion appears to be mediated via the increased exposure of NHE3-containing apical surface area of gill ionocytes, rather than changes in NHE3 or NKA protein abundance or localisation. Additionally, sea bass erythrocyte pH_i_ is regulated even more rapidly than blood pH (40 min), which enables them to quickly restore the affinity of Hb for O_2_, and therefore blood O_2_ transport capacity during exposure to elevated CO_2_. In conjunction, these acid–base regulatory responses will minimise the impact of pronounced and rapidly fluctuating CO_2_ in their natural environments, and so may prevent disruption of energetically costly activities such as foraging or digestion, and may make sea bass more resilient to the impacts of hypoxia and additional stressors during acute periods of hypercapnia. This is an avenue where we believe further research effort is necessary.

## References

[JEB242735C1] Baker, D. W., Matey, V., Huynh, K. T., Wilson, J. M., Morgan, J. D. and Brauner, C. J. (2009a). Complete intracellular pH protection during extracellular pH depression is associated with hypercarbia tolerance in white sturgeon, *Acipenser transmontanus*. *Am. J. Physiol. Integr. Comp. Physiol.* 296, R1868-R1880. 10.1152/ajpregu.90767.2008 19339675

[JEB242735C2] Baker, D. W., May, C. and Brauner, C. J. (2009b). A validation of intracellular pH measurements in fish exposed to hypercarbia: the effect of duration of tissue storage and efficacy of the metabolic inhibitor tissue homogenate method. *J. Fish Biol.* 75, 268-275. 10.1111/j.1095-8649.2009.02261.x 20738495

[JEB242735C3] Bass, J. J., Wilkinson, D. J., Rankin, D., Phillips, B. E., Szewczyk, N. J., Smith, K. and Atherton, P. J. (2017). An overview of technical considerations for Western blotting applications to physiological research. *Scand. J. Med. Sci. Sports* 27, 4-25. 10.1111/sms.12702 27263489 PMC5138151

[JEB242735C4] Baumann, H., Wallace, R. B., Tagliaferri, T. and Gobler, C. J. (2015). Large natural pH, CO_2_ and O_2_ fluctuations in a temperate tidal salt marsh on diel, seasonal, and interannual time scales. *Estuaries Coasts* 38, 220-231. 10.1007/s12237-014-9800-y

[JEB242735C5] Biemesderfer, D., Rutherford, P. A., Nagy, T., Pizzonia, J. H., Abu-Alfa, A. K. and Aronson, P. S. (1997). Monoclonal antibodies for high-resolution localization of NHE3 in adult and neonatal rat kidney. *Am. J. Physiol. Renal Physiol.* 273, F289-F299. 10.1152/ajprenal.1997.273.2.F289 9277590

[JEB242735C6] Blondeau-Bidet, E., Hiroi, J. and Lorin-Nebel, C. (2019). Ion uptake pathways in European sea bass *Dicentrarchus labrax*. *Gene* 692, 126-137. 10.1016/j.gene.2019.01.006 30641214

[JEB242735C7] Borges, A. V., Schiettecatte, L.-S., Abril, G., Delille, B. and Gazeau, F. (2006). Carbon dioxide in European coastal waters. *Estuar. Coast. Shelf Sci.* 70, 375-387. 10.1016/j.ecss.2006.05.046

[JEB242735C8] Boutilier, R. G., Heming, T. A. and Iwama, G. K. (1984). Appendix: physicochemical parameters for use in fish respiratory physiology. In *Fish Physiology* (ed. W. S. Hoar and D. J. Randall), pp. 403-430. Academic Press.

[JEB242735C9] Boutilier, R. G., Iwama, G. K., Heming, T. A. and Randall, D. J. (1985). The apparent pK of carbonic acid in rainbow trout blood plasma between 5 and 15°C. *Respir. Physiol.* 61, 237-254. 10.1016/0034-5687(85)90129-X 3931193

[JEB242735C110] Bradford, M. M. (1976). A rapid and sensitive method for the quantitation of microgram quantities of protein utilizing the principle of protein-dye binding. *Anal. Biochem.* 72, 248-254. 942051 10.1016/0003-2697(76)90527-3

[JEB242735C10] Brauner, C. J. and Baker, D. W. (2009). Patterns of acid–base regulation during exposure to hypercarbia in fishes. In *Cardio-Respiratory Control in Vertebrates: Comparative and Evolutionary Aspects* (ed. M. L. Glass and S. C. Wood), pp. 43-63. Berlin, Heidelberg: Springer Berlin Heidelberg.

[JEB242735C11] Brauner, C. J., Shartau, R. B., Damsgaard, C., Esbaugh, A. J., Wilson, R. W. and Grosell, M. (2019). 3 - Acid-base physiology and CO_2_ homeostasis: regulation and compensation in response to elevated environmental CO_2_. In *Carbon Dioxide* (ed. M. Grosell, P.L. Munday, A. P. Farrell and C. J. Brauner), pp. 69-132. Academic Press.

[JEB242735C12] Cai, W.-J., Hu, X., Huang, W.-J., Murrell, M. C., Lehrter, J. C., Lohrenz, S. E., Chou, W.-C., Zhai, W., Hollibaugh, J. T., Wang, Y. et al. (2011). Acidification of subsurface coastal waters enhanced by eutrophication. *Nat. Geosci* 4, 766-770. 10.1038/ngeo1297

[JEB242735C13] Cameron, J. N. and Iwama, G. K. (1987). Compensation of progressive hypercapnia in channel catfish and blue crabs. *J. Exp. Biol.* 133, 183-197. 10.1242/jeb.133.1.183

[JEB242735C14] Cameron, J. N. and Randall, D. J. (1972). The effect of increased ambient CO_2_ on arterial CO_2_ tension, CO_2_ content and pH in rainbow trout. *J. Exp. Biol.* 57, 673-680. 10.1242/jeb.57.3.673 4651664

[JEB242735C15] Chandrasekar, S., Nich, T., Tripathi, G., Sahu, N. P., Pal, A. K. and Dasgupta, S. (2014). Acclimation of brackish water pearl spot (*Etroplus suratensis*) to various salinities: relative changes in abundance of branchial Na^+^/K^+^-ATPase and Na^+^/K^+^/2Cl^−^ co-transporter in relation to osmoregulatory parameters. *Fish Physiol. Biochem.* 40, 983-996. 10.1007/s10695-013-9899-y 24482094

[JEB242735C16] Chen, X. L., Zhang, B., Chng, Y. R., Ong, J. L. Y., Chew, S. F., Wong, W. P., Lam, S. H. and Ip, Y. K. (2017). Na^+^/H^+^ Exchanger 3 is expressed in two distinct types of ionocyte, and probably augment ammonia exretion in one of them, in the gills of the climbing perch exposed to seawater. *Front. Physiol.* 8, 880. 10.3389/fphys.2017.00880 29209224 PMC5701670

[JEB242735C17] Christensen, A. K., Hiroi, J., Schultz, E. T. and McCormick, S. D. (2012). Branchial ionocyte organization and ion-transport protein expression in juvenile alewives acclimated to freshwater or seawater. *J. Exp. Biol.* 215, 642-652. 10.1242/jeb.063057 22279071

[JEB242735C18] Claiborne, J. B. and Heisler, N. (1984). Acid-base regulation and ion transfers in the Carp (*Cyprinus Carpio*) during and after exposure to environmental hypercapnia. *J. Exp. Biol.* 108, 25-43. 10.1242/jeb.108.1.25 3027233

[JEB242735C19] Claiborne, J. B. and Heisler, N. (1986). Acid-base regulation and ion transfers in the carp (*Cyprinus carpio*): pH compensation during graded long- and short-term environmental hypercapnia, and the effect of bicarbonate infusion. *J. Exp. Biol.* 126, 41-61. 10.1242/jeb.126.1.41 3027233

[JEB242735C20] Claiborne, J. B., Edwards, S. L. and Morrison-Shetlar, A. I. (2002). Acid-base regulation in fishes: cellular and molecular mechanisms. *J. Exp. Zool.* 293, 302-319. 10.1002/jez.10125 12115903

[JEB242735C21] Cooper, C. A., Whittamore, J. M. and Wilson, R. W. (2010). Ca_2_^+^-driven intestinal HCO_3_^−^ secretion and CaCO_3_ precipitation in the European flounder in vivo: influences on acid-base regulation and blood gas transport. *Am. J. Physiol. Integr. Comp. Physiol.* 298, R870-R876. 10.1152/ajpregu.00513.2009 PMC2853387 20130227

[JEB242735C22] Cossins, A. R. and Richardson, P. A. (1985). Adrenalin-induced Na^+^/H^+^ exchange in’ trout erythrocytes and its effects upon oxygen-carrying capacity. *J. Exp. Biol.* 118, 229-246. 10.1242/jeb.118.1.229

[JEB242735C23] Crocker, C. E. and Cech, J. J. (1997). Effects of environmental hypoxia on oxygen consumption rate and swimming activity in juvenile white sturgeon, *Acipenser transmontanus*, in relation to temperature and life intervals. *Environ. Biol. Fishes* 50, 383-389. 10.1023/A:1007362018352

[JEB242735C24] Crocker, C. E. and Cech, J. J. Jr. (1998). Effects of hypercapnia on blood-gas and acid-base status in the white sturgeon, *Acipenser transmontanus*. *J. Comp. Physiol. B* 168, 50-60. 10.1007/s003600050120

[JEB242735C25] Damsgaard, C., Gam, L. T. H., Tuong, D. D., Thinh, P. V., Huong Thanh, D. T., Wang, T. and Bayley, M. (2015). High capacity for extracellular acid-base regulation in the air-breathing fish *Pangasianodon hypophthalmus*. *J. Exp. Biol.* 218, 1290-1294. 10.1242/jeb.117671 25792754

[JEB242735C26] Dickson, A. G. and Millero, F. J. (1987). A comparison of the equilibrium constants for the dissociation of carbonic acid in seawater media. *Deep Sea Res. Part A. Oceanogr. Res. Pap.* 34, 1733-1743. 10.1016/0198-0149(87)90021-5

[JEB242735C27] Dickson, A. G., Sabine, C. L. and Christian, J. R. (2007). *Guide to Best Practices for Ocean CO2 Measurements*. North Pacific Marine Science Organization.

[JEB242735C28] Doney, S. C., Fabry, V. J., Feely, R. A. and Kleypas, J. A. (2009). Ocean acidification: the other CO_2_ problem. *Ann. Rev. Mar. Sci.* 1, 169-192. 10.1146/annurev.marine.010908.163834 21141034

[JEB242735C109] Doney, S. C., Ruckelshaus, M., Emmett Duffy, J., Barry, J. P., Chan, F., English, C. A., Galindo, H. M., Grebmeier, J. M., Hollowed, A. B., Knowlton, N. et al. (2011). Climate Change Impacts on Marine Ecosystems. *Ann. Rev. Mar. Sci.* 4, 11-37. 10.1146/annurev-marine-041911-111611 22457967

[JEB242735C29] Doyle, T. K., Haberlin, D., Clohessy, J., Bennison, A. and Jessopp, M. (2017). Localised residency and inter-annual fidelity to coastal foraging areas may place sea bass at risk to local depletion. *Sci. Rep.* 7, 45841. 10.1038/srep45841 PMC5379199 28374772

[JEB242735C30] Duarte, C. M., Hendriks, I. E., Moore, T. S., Olsen, Y. S., Steckbauer, A., Ramajo, L., Carstensen, J., Trotter, J. A. and McCulloch, M. (2013). Is ocean acidification an open-ocean syndrome? understanding anthropogenic impacts on seawater pH. *Estuaries Coasts* 36, 221-236. 10.1007/s12237-013-9594-3

[JEB242735C31] Dutta, S., Ray, S. K., Pailan, G. H., Suresh, V. R. and Dasgupta, S. (2019). Alteration in branchial NKA and NKCC ion-transporter expression and ionocyte distribution in adult hilsa during up-river migration. *J. Comp. Physiol. B* 189, 69-80. 10.1007/s00360-018-1193-y 30483930

[JEB242735C32] Eddy, F. B., Lomholt, J. P., Weber, R. E. and Johansen, K. (1977). Blood respiratory properties of rainbow trout (*Salmo gairdneri*) kept in water of high CO_2_ tension. *J. Exp. Biol.* 67, 37-47. 10.1242/jeb.67.1.37 19548

[JEB242735C33] Edwards, S. L., Wall, B. P., Morrison-Shetlar, A., Sligh, S., Weakley, J. C. and Claiborne, J. B. (2005). The effect of environmental hypercapnia and salinity on the expression of NHE-like isoforms in the gills of a euryhaline fish (*Fundulus heteroclitus*). *J. Exp. Zool.* 303A, 464-475. 10.1002/jez.a.175 15880778

[JEB242735C34] Ern, R. and Esbaugh, A. J. (2016). Hyperventilation and blood acid–base balance in hypercapnia exposed red drum (*Sciaenops ocellatus*). *J. Comp. Physiol. B* 186, 447-460. 10.1007/s00360-016-0971-7 26922790

[JEB242735C35] Esbaugh, A. J. (2017). Physiological implications of ocean acidification for marine fish: emerging patterns and new insights. *J. Comp. Physiol. B* 188, 1-13. 10.1007/s00360-017-1105-6 28547292

[JEB242735C36] Esbaugh, A. J., Heuer, R. and Grosell, M. (2012). Impacts of ocean acidification on respiratory gas exchange and acid-base balance in a marine teleost, *Opsanus beta*. *J. Comp. Physiol. B.* 182, 921-934. 10.1007/s00360-012-0668-5 22581071

[JEB242735C37] Evans, D. H., Piermarini, P. M. and Choe, K. P. (2005). The multifunctional fish gill: dominant site of gas exchange, osmoregulation, acid-base regulation, and excretion of nitrogenous waste. *Physiol. Rev.* 85, 97-177. 10.1152/physrev.00050.2003 15618479

[JEB242735C38] Feely, R. A., Sabine, C. L., Hernandez-Ayon, J. M., Ianson, D. and Hales, B. (2008). Evidence for upwelling of corrosive & “acidified” water onto the continental shelf. *Science (80)* 320, 1490-1492. 10.1126/science.1155676 18497259

[JEB242735C39] Frankignoulle, M. and Borges, A. V. (2001). Direct and Indirect *p*CO_2_ measurements in a wide range of *p*CO_2_ and salinity values (The Scheldt Estuary). *Aquat. Geochemistry* 7, 267-273. 10.1023/A:1015251010481

[JEB242735C40] Frommel, A. Y., Kwan, G. T., Prime, K. J., Tresguerres, M., Lauridsen, H., Val, A. L., Gonçalves, L. U. and Brauner, C. J. (2021). Changes in gill and air-breathing organ characteristics during the transition from water- to air-breathing in juvenile *Arapaima gigas*. *J Exp. Zool.* 335A, 801-813. 10.1002/jez.2456 33819380

[JEB242735C41] Goss, G. G., Laurent, P. and Perry, S. F. (1992a). Evidence for a morphological component in acid-base regulation during environmental hypercapnia in the brown bullhead (*Ictalurus nebulosus*). *Cell Tissue Res.* 268, 539-552. 10.1007/BF00319161 1628310

[JEB242735C42] Goss, G. G., Perry, S. F., Wood, C. M. and Laurent, P. (1992b). Mechanisms of ion and acid-base regulation at the gills of freshwater fish. *J. Exp. Zool.* 263, 143-159. 10.1002/jez.1402630205 1500882

[JEB242735C43] Goss, G. G., Laurent, P. and Perry, S. F. (1994). Gill morphology during hypercapnia in brown bullhead (*Ictalurus nebulosus*): role of chloride cells and pavement cells in acid-base regulation. *J. Fish Biol.* 45, 705-718. 10.1111/j.1095-8649.1994.tb00938.x

[JEB242735C44] Gwozdz, T. and Dorey, K. (2017). Chapter 6 – western blot. In *Basic Science Methods for Clinical Researchers* (ed. M. Jalali, F. Y. L. Saldanha and M. Jalali), pp. 99-117. Academic Press.

[JEB242735C45] Harter, T. S., Clifford, A. M. and Tresguerres, M. (2021). Adrenergically induced translocation of red blood cell β-adrenergic sodium-proton exchangers has ecological for hypoxic and hypercapnic white seabass. *Am. J. Physiol. Integr. Comp. Physiol.* 321, R655-R671. 10.1152/ajpregu.00175.2021 34494485

[JEB242735C46] Hayashi, M., Kita, J. and Ishimatsu, A. (2004). Acid-base responses to lethal aquatic hypercapnia in three marine fishes. *Mar. Biol.* 144, 153-160. 10.1007/s00227-003-1172-y

[JEB242735C47] Hayashi, M., Kikkawa, T. and Ishimatsu, A. (2013). Morphological changes in branchial mitochondria-rich cells of the teleost *Paralichthys olivaceus* as a potential indicator of CO_2_ impacts. *Mar. Pollut. Bull.* 73, 409-415. 10.1016/j.marpolbul.2013.06.034 23838416

[JEB242735C48] Hiroi, J. and McCormick, S. D. (2012). New insights into gill iomocyte and ion transporter function in euryhaline and diadromous fish. *Resp. Physiol. Neurobiol.* 184, 257-268. 10.1016/j.resp.2012.07.019 22850177

[JEB242735C49] Hofmann, G. E., Smith, J. E., Johnson, K. S., Send, U., Levin, L. A., Micheli, F., Paytan, A., Price, N. N., Peterson, B., Takeshita, Y. et al. (2011). High-frequency dynamics of ocean pH: a multi-ecosystem comparison. *PLoS ONE* 6, e28983. 10.1371/journal.pone.0028983 22205986 PMC3242773

[JEB242735C112] IPCC (2014). *Climate Change 2014: Synthesis Report. Contribution of Working Groups I, II and III to the Fifth Assessment Report of the Intergovernmental Panel on Climate Change*. Geneva, Switzerland.

[JEB242735C51] Kumai, Y. and Perry, S. F. (2012). Mechanisms and regulation of Na^+^ uptake by freshwater fish. *Respir. Physiol. Neurobiol.* 184, 249-256. 10.1016/j.resp.2012.06.009 22698881

[JEB242735C52] Kwan, G. T., Wexler, J. B., Wegner, N. C. and Tresguerres, M. (2019). Ontogenetic changes in cutaneous and branchial ionocytes and morphology in yellowfin tuna (*Thunnus albacares*) larvae. *J. Comp. Physiol. B* 189, 81-95. 10.1007/s00360-018-1187-9 30357584

[JEB242735C53] Kwan, G. T., Smith, T. R. and Tresguerres, M. (2020). Immunological characterization of two types of ionocytes in the inner ear epithelium of Pacific Chub Mackerel (*Scomber japonicus*). *J. Comp. Physiol. B* 190, 419-431. 10.1007/s00360-020-01276-3 32468089

[JEB242735C54] Kwan, G. T., Shen, S. G., Drawbridge, M., Chekley, D. M., Jr and Tresguerres, M. (2021). Ion-transporting capacity and aerobic respiration of larval white seabass (*Atractoscion nobilis*) may be resilient to ocean acidification conditions. *Sci. Total Environ.* 791, 148285. 10.1016/j.scitotenv.2021.148285 34126476

[JEB242735C55] Larsen, B. K. and Jensen, F. B. (1997). Influence of ionic composition on acid-base regulation in rainbow trout (*Oncorhynchus mykiss*) exposed to environmental hypercapnia. *Fish Physiol. Biochem.* 16, 157-170. 10.1007/BF00004672

[JEB242735C56] Larsen, B. K., Pörtner, H.-O. and Jensen, F. B. (1997). Extra- and intracellular acid-base balance and ionic regulation in cod (*Gadus morhua*) during combined and isolated exposures to hypercapnia and copper. *Mar. Biol.* 128, 337-346. 10.1007/s002270050099

[JEB242735C57] Lebovitz, R. M., Takeyasu, K. and Fambrough, D. M. (1989). Molecular characterization and expression of the (Na^+^+K^+^)-ATPase alpha-subunit in *Drosophila melanogaster*. *EMBO J.* 8, 193-202. 10.1002/j.1460-2075.1989.tb03364.x 2540956 PMC400789

[JEB242735C58] Lee, K.-S., Kita, J. and Ishimatsu, A. (2003). Effects of lethal levels of environmental hypercapnia on cardiovascular and blood-gas status in yellowtail, *Seriola quinqueradiata*. *Zoolog. Sci.* 20, 417-422. 10.2108/zsj.20.417 12719643

[JEB242735C59] Leino, R. L. and McCormick, J. H. (1984). Morphological and morphometrical changes in chloride cells of the gills of *Pimephales promelas* after chronic exposure to acid water. *Cell Tissue Res.* 236, 121-128. 10.1007/BF00216521 6713499

[JEB242735C60] Lewis, C., Clemow, K. and Holt, W. V. (2013). Metal contamination increases the sensitivity of larvae but not gametes to ocean acidification in the polychaete *Pomatoceros lamarckii* (Quatrefages). *Mar. Biol.* 160, 2089-2101. 10.1007/s00227-012-2081-8

[JEB242735C61] McCormick, S. D., Regish, A. M. and Christensen, A. K. (2009). Distinct freshwater and seawater isoforms of Na^+^/K^+^-ATPase in gill chloride cells of Atlantic salmon. *J. Exp. Biol.* 212, 3994-4001. 10.1242/jeb.037275 19946077

[JEB242735C62] McDonald, D. G. and Wood, C. M. (1981). Branchial and renal acid and ion fluxes in the Rainbow Trout, *Salmo gairdneri*, at Low Environmental pH. *J. Exp. Biol.* 93, 101-118. 10.1242/jeb.93.1.101 7452134

[JEB242735C63] Melzner, F., Thomsen, J., Koeve, W., Oschlies, A., Gutowska, M. A., Bange, H. W., Hansen, H. P. and Körtzinger, A. (2013). Future ocean acidification will be amplified by hypoxia in coastal habitats. *Mar. Biol.* 160, 1875-1888. 10.1007/s00227-012-1954-1

[JEB242735C64] Middlemiss, K. L., Urbina, M. A. and Wilson, R. W. (2016). Effects of seawater alkalinity on calcium and acid–base regulation in juvenile European lobster (*Homarus gammarus*) during a moult cycle. *Comp. Biochem. Physiol. Part A Mol. Integr. Physiol.* 193, 22-28. 10.1016/j.cbpa.2015.12.002 26691956

[JEB242735C65] Montgomery, D. W., Simpson, S. D., Engelhard, G. H., Birchenough, S. N. R. and Wilson, R. W. (2019). Rising CO_2_ enhances hypoxia tolerance in a marine fish. *Sci. Rep.* 9, 15152. 10.1038/s41598-019-51572-4 31641181 PMC6805886

[JEB242735C66] Motulsky, H. J. and Brown, R. E. (2006). Detecting outliers when fitting data with nonlinear regression – a new method based on robust nonlinear regression and the false discovery rate. *BMC Bioinformatics* 7, 123. 10.1186/1471-2105-7-123 16526949 PMC1472692

[JEB242735C67] Nadler, L. E., Bengston, E., Eliason, E. J., Hassibi, C., Helland-Riise, S. H., Johansen, I. B., Kwan, G. T., Tresguerres, M., Turner, A. V., Weinersmith, K. L. et al. (2021). A brain-infecting parasite impacts host metabolism both during exposure and after infection is established. *Funct. Ecol.* 35, 105-116. 10.1111/1365-2435.13695

[JEB242735C68] Nikinmaa, M. (2012). *Vertebrate Red Blood Cells: Adaptations of Function to Respiratory Requirements*. Springer Science & Business Media.

[JEB242735C69] Nikinmaa, M. and Tufts, B. L. (1989). Regulation of acid and ion transfer across the membrane of nucleated erythrocytes. *Can. J. Zool.* 67, 3039-3045. 10.1139/z89-427

[JEB242735C70] Oellermann, M., Pörtner, H.-O. and Mark, F. C. (2014). Simultaneous high-resolution pH and spectrophotometric recordings of oxygen binding in blood microvolumes. *J. Exp. Biol.* 217, 1430-1436. 10.1242/jeb.092726 24436387

[JEB242735C111] Orr, J. C., Fabry, V. J., Aumont, O., Bopp, L., Doney, S. C., Feely, R. A., Gnanadesikan, A., Gruber, N., Ishida, A. and Joos, F. (2005). Anthropogenic ocean acidification over the twenty-first century and its impact on calcifying organisms. *Nature* 437, 681-686. 16193043 10.1038/nature04095

[JEB242735C71] Pan, T.-C. F., Applebaum, S. L. and Manahan, D. T. (2015). Experimental ocean acidification alters the allocation of metabolic energy. *Proc. Natl. Acad. Sci. USA* 112, 4696-4701. 10.1073/pnas.1416967112 25825763 PMC4403215

[JEB242735C72] Parks, S. K., Tresguerres, M. and Goss, G. G. (2007). Blood and gill responses to HCl infusions in the Pacific hagfish (*Eptatretus stoutii*). *Can. J. Zool.* 85, 855-862. 10.1139/Z07-068

[JEB242735C73] Parks, S. K., Tresguerres, M. and Goss, G. G. (2008). Theoretical considerations underlying Na^+^ uptake mechanisms in freshwater fishes. *Comp. Biochem. Physiol. Part C Toxicol. Pharmacol.* 148, 411-418. 10.1016/j.cbpc.2008.03.002 18420463

[JEB242735C74] Perry, S. F. (1982). The regulation of hypercapnic acidosis in two Salmonids, the freshwater trout (*Salmo gairdneri*) and the seawater salmon (*Onchorynchus kisutch*). *Mar. Behav. Physiol.* 9, 73-79. 10.1080/10236248209378584

[JEB242735C75] Perry, S. F. and Gilmour, K. M. (2006). Acid–base balance and CO_2_ excretion in fish: Unanswered questions and emerging models. *Respir. Physiol. Neurobiol.* 154, 199-215. 10.1016/j.resp.2006.04.010 16777496

[JEB242735C76] Perry, S. F. and Kinkead, R. (1989). The role of catecholamines in regulating arterial oxygen content during acute hypercapnic acidosis in rainbow trout (*Salmo gairdneri*). *Respir. Physiol.* 77, 365-377. 10.1016/0034-5687(89)90123-0 2781171

[JEB242735C77] Perry, S. F. and McKendry, J. E. (2001). The relative roles of external and internal CO_2_ versus H^+^ in eliciting the cardiorespiratory responses of *Salmo salar* and *Squalus acanthias* to hypercarbia. *J. Exp. Biol.* 204, 3963-3971. 10.1242/jeb.204.22.3963 11807114

[JEB242735C78] Perry, S. F., Haswell, M. S., Randall, D. J. and Farrell, A. P. (1981). Branchial ionic uptake and acid-base regulation in the rainbow trout, *Salmo Gairdneri**.* *J. Exp. Biol.* 92, 289-303. 10.1242/jeb.92.1.289

[JEB242735C79] Perry, S. F., Fritsche, R., Hoagland, T. M., Duff, D. W. and Olson, K. R. (1999). The control of blood pressure during external hypercapnia in the rainbow trout (*Oncorhynchus mykiss*). *J. Exp. Biol.* 202, 2177-2190. 10.1242/jeb.202.16.2177 10409489

[JEB242735C80] Pierrot, D., Lewis, E. and Wallace, D. W. R. (2006). CO2SYS DOS program developed for CO2 system calculations. *ORNL/CDIAC-105*. Oak Ridge, Tennesee: Oak Ridge National Laboratory.

[JEB242735C81] Roa, J. N., Munévar, C. L. and Tresguerres, M. (2014). Feeding induces translocation of vacuolar proton ATPase and pendrin to the membrane of leopard shark (*Triakis semifasciata*) mitochondrion-rich gill cells. *Comp. Biochem. Physiol. A Mol. Integr. Physiol.* 174, 29-37. 10.1016/j.cbpa.2014.04.003 24746982 PMC6278952

[JEB242735C82] Rogers, N. J. (2015). The Respiratory and Gut Physiology of Fish: Response to Environmental Change. *PhD Thesis*, University of Exeter, Exeter, Devon.

[JEB242735C83] Saderne, V., Fietzek, P. and Herman, P. M. J. (2013). Extreme variations of *p*CO_2_ and pH in a macrophyte meadow of the baltic sea in summer: evidence of the effect of photosynthesis and local upwelling. *PLoS ONE* 8, e62689. 10.1371/journal.pone.0062689 23626849 PMC3633870

[JEB242735C84] Salisbury, J., Green, M., Hunt, C. and Campbell, J. (2008). Coastal acidification by rivers: a threat to shellfish? *Eos, Trans. Am. Geophys. Union* 89, 513. 10.1029/2008EO500001

[JEB242735C85] Schindelin, J., Arganda-Carreras, I., Frise, E., Kaynig, V., Longair, M., Pietzsch, T., Preibisch, S., Rueden, C., Saalfeld, S., Schmid, B. et al. (2012). Fiji: an open-source platform for biological-image analysis. *Nat. Methods* 9, 676-682. 10.1038/nmeth.2019 22743772 PMC3855844

[JEB242735C86] Seo, M. Y., Mekuchi, M., Teranishi, K. and Kaneko, T. (2013). Expression of ion transporters in gill mitochondrion-rich cells in Japanese eel acclimated to a wide range of environmental salinity. *Comp. Biochem. Physiol. A Mol. Integr. Physiol.* 166, 323-332. 10.1016/j.cbpa.2013.07.004 23838143

[JEB242735C87] Shartau, R. B., Baker, D. W., Harter, T. S., Aboagye, D. L., Allen, P. J., Val, A. L., Crossley, D. A., Kohl, Z. F., Hedrick, M. S., Damsgaard, C. et al. (2020). Preferential intracellular pH regulation is a common trait amongst fishes exposed to high environmental CO_2_. *J. Exp. Biol.* 223, jeb208868. 10.1242/jeb.208868 32127382

[JEB242735C88] Smatresk, N. J. and Cameron, J. N. (1982). Respiration and acid-base physiology of the spotted gar, a bimodal breather: II. Responses to temperature change and hypercapnia. *J. Exp. Biol.* 96, 281-293. 10.1242/jeb.96.1.281

[JEB242735C89] Sunda, W. G. and Cai, W.-J. (2012). Eutrophication induced CO_2_-acidification of subsurface coastal waters: interactive effects of temperature, salinity, and atmospheric *p*CO_2_. *Environ. Sci. Technol.* 46, 10651-10659. 10.1021/es300626f 22889106

[JEB242735C90] Thomas, S. and Perry, S. F. (1992). Control and consequences of adrenergic activation of red blood cell Na^+^/H^+^ exchange on blood oxygen and carbon dioxide transport in fish. *J. Exp. Zool.* 263, 160-175. 10.1002/jez.1402630206 1323642

[JEB242735C91] Toews, D. P., Holeton, G. F. and Heisler, N. (1983). Regulation of the acid-base status during environmental hypercapnia in the marine teleost fish *Conger conger*. *J. Exp. Biol.* 107, 9-20. 10.1242/jeb.107.1.9 6668465

[JEB242735C92] Tovey, K. J. and Brauner, C. J. (2018). Effects of water ionic composition on acid–base regulation in rainbow trout, during hypercarbia at rest and during sustained exercise. *J. Comp. Physiol. B* 188, 295-304. 10.1007/s00360-017-1129-y 29067494

[JEB242735C93] Tresguerres, M. and Hamilton, T. J. (2017). Acid base physiology, neurobiology and behaviour in relation to CO_2_-induced ocean acidification. *J. Exp. Biol.* 220, 2136-2148. 10.1242/jeb.144113 28615486

[JEB242735C94] Tresguerres, M., Katoh, F., Fenton, H., Jasinska, E. and Goss, G. G. (2005). Regulation of branchial V-H^+^-ATPase, Na^+^/K^+^-ATPase and NHE2 in response to acid and base infusions in the Pacific spiny dogfish (*Squalus acanthias*). *J. Exp. Biol.* 208, 345-354. 10.1242/jeb.01382 15634853

[JEB242735C95] Tresguerres, M., Parks, S. K., Katoh, F. and Goss, G. G. (2006). Microtubule-dependent relocation of branchial V-H^+^-ATPase to the basolateral membrane in the Pacific spiny dogfish (*Squalus acanthias*): a role in base secretion. *J. Exp. Biol.* 209, 599-609. 10.1242/jeb.02059 16449555

[JEB242735C96] Tresguerres, M., Parks, S. K. and Goss, G. G. (2007a). Recovery from blood alkalosis in the Pacific hagfish (*Eptatretus stoutii*): Involvement of gill V–H^+^–ATPase and Na^+^/K^+^–ATPase. *Comp. Biochem. Physiol. A Mol. Integr. Physiol.* 148, 133-141. 10.1016/j.cbpa.2007.03.032 17512231

[JEB242735C97] Tresguerres, M., Parks, S. K., Wood, C. M. and Goss, G. G. (2007b). V-H^+^-ATPase translocation during blood alkalosis in dogfish gills: interaction with carbonic anhydrase and involvement in the postfeeding alkaline tide. *Am. J. Physiol. Integr. Comp. Physiol.* 292, R2012-R2019. 10.1152/ajpregu.00814.2006 17204588

[JEB242735C98] Tresguerres, M., Milsom, W. K. and Perry, S. F. (2019). 2 - CO_2_ and acid-base sensing. In *Carbon Dioxide* (ed. M. Grossell, P. L. Munday, A. P. Farrell and C. J. Brauner), pp. 33-68. Academic Press.

[JEB242735C99] Tseng, Y.-C., Hu, M. Y., Stumpp, M., Lin, L.-Y., Melzner, F. and Hwang, P.-P. (2013). CO_2_-driven seawater acidification differentially affects development and molecular plasticity along life history of fish (*Oryzias latipes*). *Comp. Biochem. Physiol. A Mol. Integr. Physiol.* 165, 119-130. 10.1016/j.cbpa.2013.02.005 23416137

[JEB242735C100] Verdouw, H., Van Echteld, C. J. A. and Dekkers, E. M. J. (1978). Ammonia determination based on indophenol formation with sodium salicylate. *Water Res.* 12, 399-402. 10.1016/0043-1354(78)90107-0

[JEB242735C101] Vermette, M. G. and Perry, S. F. (1988). Adrenergic involvement in blood oxygen transport and acid-base balance during hypercapnic acidosis in the Rainbow Trout, *Salmo gairdneri*. *J. Comp. Physiol. B* 158, 107-115. 10.1007/BF00692734

[JEB242735C102] Wallace, R. B., Baumann, H., Grear, J. S., Aller, R. C. and Gobler, C. J. (2014). Coastal ocean acidification: The other eutrophication problem. *Estuar. Coast. Shelf Sci.* 148, 1-13. 10.1016/j.ecss.2014.05.027

[JEB242735C103] Wells, R. M. G. (2009). Chapter 6 Blood-gas transport and hemoglobin function: adaptations for functional and environmental hypoxia. In *Fish Physiology* (ed. J. G. Richards, A. P. Farrell and C. J. Brauner), pp. 255-299. Academic Press.

[JEB242735C104] Wilson, R. W. and Grosell, M. (2003). Intestinal bicarbonate secretion in marine teleost fish—source of bicarbonate, pH sensitivity, and consequences for whole animal acid–base and calcium homeostasis. *Biochim. Biophys. Acta - Biomembr.* 1618, 163-174. 10.1016/j.bbamem.2003.09.014 14729153

[JEB242735C105] Wood, C. M., McDonald, D. G. and McMahon, B. R. (1982). The Influence of Experimental Anaemia on Blood Acid-Base Regulation In Vivo and In Vitro in the Starry Flounder (*Platichthys Stellatus*) and the Rainbow Trout (*Salmo Gairdneri*). *J. Exp. Biol.* 96, 221-237. 10.1242/jeb.96.1.221

[JEB242735C106] Wright, P. A., Wood, C. M., Hiroi, J. and Wilson, J. M. (2016). (Uncommon) Mechanism of branchial ammonia excretion in the common Carp (*Cyprinus carpio*) in response to environmentally induced metabolic acidosis. *Physiol. Biochem. Zool.* 89, F289-F299. 10.1086/683990 27082522

[JEB242735C107] Zadunaisky, J. A. (1996). Chloride cells and osmoregulation. *Kidney Int.* 49, 1563-1567. 10.1038/ki.1996.225 8743455

[JEB242735C108] Zeidler, R. and Kim, H. D. (1977). Preferential hemolysis of postnatal calf red cells induced by internal alkalinization. *J. Gen. Physiol.* 70, 385-401. 10.1085/jgp.70.3.385 19557 PMC2228469

